# Quantification of Stereochemical Communication in Metal–Organic Assemblies

**DOI:** 10.1002/anie.201602968

**Published:** 2016-06-02

**Authors:** Ana M. Castilla, Mark A. Miller, Jonathan R. Nitschke, Maarten M. J. Smulders

**Affiliations:** ^1^ Laboratory of Organic ChemistryWageningen UniversityStippeneng 46708 WEWageningenThe Netherlands; ^2^ Department of ChemistryUniversity of CambridgeCambridgeCB2 1EWUK; ^3^ Department of ChemistryDurham UniversitySouth RoadDurhamDH1 3LEUK

**Keywords:** cage compounds, diastereoselectivity, statistical mechanics, stereochemical communication, supramolecular chemistry

## Abstract

The derivation and application of a statistical mechanical model to quantify stereochemical communication in metal–organic assemblies is reported. The factors affecting the stereochemical communication within and between the metal stereocenters of the assemblies were experimentally studied by optical spectroscopy and analyzed in terms of a free energy penalty per “incorrect” amine enantiomer incorporated, and a free energy of coupling between stereocenters. These intra‐ and inter‐vertex coupling constants are used to track the degree of stereochemical communication across a range of metal–organic assemblies (employing different ligands, peripheral amines, and metals); temperature‐dependent equilibria between diastereomeric cages are also quantified. The model thus provides a unified understanding of the factors that shape the chirotopic void spaces enclosed by metal–organic container molecules.

By virtue of their hollow interiors, metal–organic container molecules^1^ offer great potential for a range of applications,^2^ including guest binding and separation, cavity‐controlled catalysis, and stabilization of reactive intermediates. In contrast to the many enantioselective transformations catalyzed inside an enzyme's (chiral) active site, in synthetic hosts the role of stereochemistry^3^ has received little attention, with the focus mostly on controlling the size of the host cavity to steer guest binding or catalysis.^4^ Reports addressing stereochemistry in metal–organic assemblies have so far mainly dealt with the synthesis of enantiopure cages,^5^ (interconverting) diastereomeric species,^6^ stereochemical switches,^7^ enantioselective catalysis,^8^ or guest binding.^9^

To emulate the enantioselectivity displayed by enzymes, insights into the conditions under which chiral ligands induce the formation of an enantiopure metal–organic self‐assembled capsule are required. Such rules can guide the design of new container molecules offering enantioselective guest binding or catalysis, and may have implications for the understanding of the origin of biological homochirality.^10^ A quantitative analysis based on statistical mechanics has proven useful in the description of chiral amplification in covalent and supramolecular polymers.^11^

The present work provides a quantitative description of the degree of stereochemical information transfer within discrete metal–organic cages. Building upon the pioneering work of Piguet on quantifying subtle thermodynamic effects in the self‐assembly of polynuclear complexes,^12^ we develop a simple statistical mechanical model that quantifies the effects of various factors, such as the choice of metal, chiral residue, ligand length, and temperature. We start with the phenomenon of amplification of stereochemical information as previously observed in a Fe^II^_4_L_6_ cage with a strong preference to have all metal centers with the same all‐Δ configuration.^13^ We then examine this phenomenon in related tetrahedral cages with different metals, ligand lengths, or geometries (Figure 1). We also apply the model to the temperature‐dependent diastereomer distributions in tetrahedral cages with weaker stereochemical coupling between metal centers.

**Figure 1:**
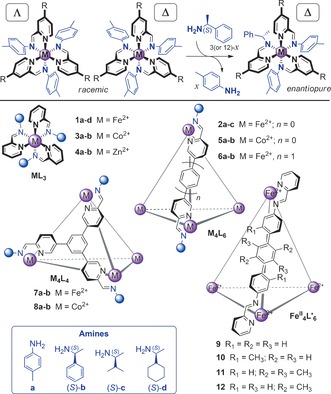
Top: Chiral induction through subcomponent substitution (R=H or a bridge within a connecting ligand). Bottom: Overview of the metal–organic assemblies studied herein.

Sergeant‐and‐soldiers experiments (Figure 1, top), involving the substitution of residues of achiral amine **a** within a racemic Fe^II^_4_L_6_ cage (**2 a**; Figure 1, bottom) with increasing amounts of a more nucleophilic enantiopure amine (*S*)‐**b**, resulted in the quantitative induction of a single stereochemical configuration at all Fe^II^ centers before 100 % (12 equiv) of the chiral amine was added, as monitored by the chiroptical response.^13^ This effect was shown to be enhanced in the cage with respect to a related mononuclear complex (**1 a**) as a result of stereochemical coupling between metal centers in the cage.

To devise a statistical model to quantify this effect, we separate the two ways in which stereochemical information can be amplified in multinuclear structures. First, at each metal center, intra‐vertex amplification can manifest itself when fewer than three chiral amine residues suffice to quantitatively induce a single Δ or Λ stereoconfiguration. Second, inter‐vertex communication: the mechanical connection between metal centers by rigid ligands allows stereochemical information to be relayed between vertices in the framework.^14^ As a result, the configuration at one metal center can influence or even dictate the configuration of its neighbors. The resulting model is a finite Ising system with quenched field disorder controlled by the distribution of chiral amines. Related models^15^ have been used to describe the binding of ions to polyelectrolytes15a, 15b and cooperativity in supramolecular chemistry.15c

We consider first the case of a mononuclear ML_3_ complex which we treat as a two‐state system with Δ and Λ states, where Δ is the preferred configuration for all (*S*)‐chiral amines in this work.^13^ Taking the Δ state as reference, an (*S*)‐amine attached to a Λ center incurs a free energy penalty, denoted *f*_1_, in units of the thermal energy *k*_B_ *T* (where *k*_B_ is the Boltzmann constant). The *f*_1_ value quantifies the strength of coupling between carbon and metal stereocenters. We treat each amine in a given metal coordination sphere as acting independently, and we take the probability of substitution as equal for all amines in the system.

For tetrahedral cages, we treat each of the four metal centers as a two‐state system, as in the mononuclear case. Now, however, there is also inter‐vertex communication, as a result of the preference of a ligand to have the same stereoconfiguration at its two ends in a given structure. Taking the ΔΔ and ΛΛ states of a ligand as the reference, we quantify the inter‐vertex stereochemical coupling through the parameter *f*_2_, defined as the free energy of the ΔΛ and ΛΔ states, divided by *k*_B_ *T*. The total dimensionless free energy of a given tetrahedral complex is a multiple of *f*_1_ plus a multiple of *f*_2_ depending on the number and location of chiral amines, and the stereoconfiguration at each of its metal centers.

A Boltzmann‐weighted average over all stereoconfigurations yields the overall fraction *x*_Δ_ of Δ centers in an equilibrium solution of the cages (see Section S3.1 in the Supporting Information). The value of *x*_Δ_ depends on the parameters *f*_1_ and *f*_2_, providing a physical interpretation of the populations of Δ and Λ centers observed in experiments. For given values of *f*_1_ and *f*_2_, the model predicts how the excess chirality (i.e. the relative excess of Δ over Λ metal centers) increases with the fraction (*s*) of substituted amine. Plots of chiral excess as a function of *s* approach a limiting form as the *f*_1_ and *f*_2_ values become large, that is, the shape of the curve eventually becomes insensitive to the precise values of *f*_1_ and *f*_2_ (see Section S3.2).

We first apply the model to analyze sergeant‐and‐soldiers experiments,^13^ which started either with a racemic Fe^II^L_3_ complex (**1 a**), or with the racemic cage Fe^II^_4_L_6_ (**2 a**), and to which different amounts of (*S*)‐amine were added (see Section S2 in the Supporting Information). We examined three chiral amines, (*S*)‐**b**,^13^ (*S*)‐**c**, and (*S*)‐**d** 16 (Figure 1, bottom), to differentiate between the abilities of the amines to induce a single‐metal stereochemical configuration, as expressed by the *f*_1_ value. Figure 2 A shows how the excess chirality, as probed by circular dichroism, varied with the amount of added amine. In these plots, both the experimental and fitted curves have been renormalized to 1 at a chiral amine concentration of 100 % (see Section S3.3). Values of *f*_1_ were obtained by least‐squares fitting to data from experiments with the Fe^II^L_3_ complexes (Figure S1). These values were then fixed and used in a second fit to the experimental data obtained for the related Fe^II^_4_L_6_ cages (Figure S4), to obtain an *f*_2_ value for each cage (Figure 2 E).

**Figure 2:**
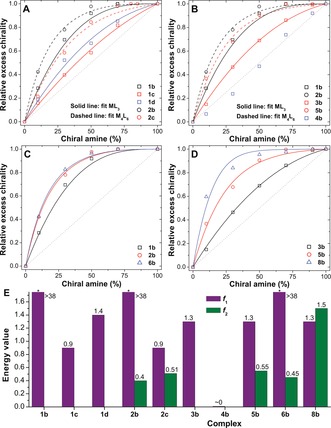
A–D) Sergeant‐and‐soldiers sensitivity plots of excess chirality (experimental data points and fitted curves) versus added (*S*)‐amine. Plots in A) are for the addition of (*S*)‐**b**, (*S*)‐**c**, or (*S*)‐**d** to Fe^II^L_3_ or Fe^II^_4_L_6_ complexes (to form complexes **1 b**–**d** and **2 b**, **c**); B) show the addition of (*S*)‐**b** to an ML_3_ or M_4_L_6_ complex (M=Fe^II^, Co^II^, or Zn^II^); C) show the addition of (*S*)‐**b** to Fe^II^L_3_, Fe^II^_4_L_6_, and Fe^II^_4_L′_6_ complexes; D) show the addition of (*S*)‐**b** to Co^II^L_3_, Co^II^_4_L_6_, and Co^II^_4_L_4_ complexes. E) Energy values for all complexes in MeCN (*: value off‐scale).

The model accounts well for the shape of the experimental curves (Figure 2 A). The *f*_1_ values of 38, 0.90, and 1.4 obtained for amines (*S*)‐**b**, (*S*)‐**c**, and (*S*)‐**d**, respectively, highlight the much stronger ability of amine (*S*)‐**b** than amine (*S*)‐**c** in controlling the configuration at the metal center. The free energy penalty of 38*k*_B_ *T* for (*S*)‐**b** lies in the limiting regime of a large *f*_1_ value, where the sergeant‐and‐soldiers effect is overwhelming and the chiroptical response is insensitive to the exact value (Figure S8). Between (*S*)‐**c** and (*S*)‐**d**, (*S*)‐**d** exhibited a stronger ability to control the configuration at the metal center, which we attribute to the greater bulk of the side group (cyclohexyl versus isopropyl).16a, 17 We infer that both sterics and π‐stacking effects between phenyl and pyridyl rings are responsible for the strong influence of amine (*S*)‐**b** upon the metal‐centered configuration.16a

Using the *f*_1_ values from the Fe^II^L_3_ complexes, experimental data could be fitted for the Fe^II^_4_L_6_ cages **2 b** and **2 c**; precipitation during the substitution of cage **2 a** with (*S*)‐**d** precluded sergeant‐and‐soldiers studies with this amine. In these cages the Fe^II^ vertices are held together by the same ligand so similar *f*_2_ values are expected as *f*_2_ measures the ligand's ability to mediate stereochemical communication between the individual metal centers (as explained above). Gratifyingly, the values of *f*_2_ for cages **2 b** and **2 c** of 0.40 and 0.51, respectively, are similar. We attribute the small difference between the two values to uncertainty in fitting experimental data and to small differences in cage geometry as a result of the different amines.

Next, the effect of metal choice on the degree of amplification was studied by performing substitution experiments with (*S*)‐**b** on the Co^II^ and Zn^II^‐containing analogues of the Fe^II^L_3_ complex **1 a** (namely **3 a** and **4 a**) and the Co^II^‐templated analogue of the Fe^II^_4_L_6_ cage **2 a** (namely **5 a**; for each metal the chiroptical data were normalized at a different wavelength; Figure S3). Our model correctly predicts the sharp decrease in *f*_1_ value from 38 for Fe^II^, to 1.3 for Co^II^ (**3 b**), to approximately 0 for Zn^II^ (**4 b**). Remarkably, no amplification was observed for Zn^II^: the excess chirality of the Zn^II^L_3_ complex **4 b** increased linearly as a function of added (*S*)‐**b**. These observations can be understood in terms of the increased metal–ligand distance when going from Fe^II^ through Co^II^ to Zn^II^,16a with a concomitant reduction in bond strength (see Section S5 in the Supporting Information), which in turn decreases the steric gearing of the chiral amine residues required for effective stereochemical control around the metal center.

Despite similar *f*_2_ values, because of the smaller *f*_1_ value for Co^II^, the enhancement in nonlinear effects in a M_4_L_6_ cage with respect to a ML_3_ complex is more pronounced in the case of the Co^II^‐containing structures, **3 b** and **5 b**, than for their Fe^II^‐templated analogues **1 b** and **2 b** (Figure 2 B). Because the Zn^II^‐analogue of **2 a** could not be prepared without an anionic template (see Section S1), its amplification behavior was not studied.

The effect of ligand structure on the degree of stereochemical communication within tetrahedral cages was studied by examining the substitution with the same amine ((*S*)‐**b**) of two other Fe^II^ cages: cage **6 a**,^18^ built from a longer ditopic ligand (compared to **2 a**), and cage **7** **a**, based upon a tritopic ligand.16b For cage **6 b**, the *f*_2_ value of 0.45 is only slightly higher than the value of 0.40 for **2 b**, verifying quantitatively the previous observation that linker length does not strongly affect the degree of stereochemical communication in these cages.17a

In stark contrast with the behavior of the ML_3_ complexes and M_4_L_6_ cages studied, the substitution of the Fe^II^_4_L_4_ cage **7 a** with (*S*)‐**b** was observed to occur through a cooperative imine exchange process (see Section S4), confirming the previously reported kinetic stability of this Fe^II^_4_L_4_ framework.16b Not being able to use cage **7 a** to investigate the degree of stereochemical coupling between metal centers in M_4_L_4_ structures, we turned to its Co^II^‐containing congener (**8 a**), which was not observed to undergo cooperative amine exchange (Figure S7, Figures S36–S37). For Co^II^_4_L_4_ cage **8 b**, stronger inter‐vertex communication was observed, as expressed by an *f*_2_ parameter of 1.5, which is significantly higher than the value of 0.55 for the corresponding Co^II^_4_L_6_ cage **5 b** (Figure 2 E). We infer that the tritopic ligands have a strong “gearing” effect within the rigid structure, forcing the four metal centers in the cage to adopt a homochirally pure Δ state. This strong inter‐vertex stereochemical coupling has been shown to enable stereochemical memory in a Fe^II^_4_L_4_ cage.16b

In addition to analyzing the transmission of stereochemical information in all‐Δ or all‐Λ cages, we have also applied our model to a set of racemic Fe^II^_4_L_6_ cages (**9**–**12**; Figure 1) that have been previously observed to form heterochiral species.6b These assembled from ditopic achiral ligands (that is, with *f*_1_=0) based on terphenyl linkers with different methylation patterns. Cages **9**–**12** were found to exist in solution as an equilibrium between homochiral *T* (ΔΔΔΔ/ΛΛΛΛ), heterochiral *C*_3_ (ΔΔΔΛ/ΛΛΛΔ), and achiral *S*_4_ (ΔΔΛΛ) diastereomers. Moreover, variable‐temperature NMR studies revealed these equilibria to be temperature‐dependent.

The model can be employed to make a direct prediction of universal curves for the equilibrium distribution of the three diastereomers as a function of *f*_2_ value (Figure 3). We have placed the temperature‐dependent distribution of diastereomers for cages **9**–**12** on these curves by finding the point on the *f*_2_ axis where each set of three yields fits best. There is no guarantee that an arbitrary set of three diastereomer fractions could be consistently placed on the curves. Hence, the observation that all data sets, apart from those for cage **9**, can be superimposed on the plot provides strong support for the underlying model. The behavior of the four cages is very different. Cage **11** has strongly negative values for *f*_2_ (<−2), indicating that the ligand prefers to connect two metal centers of opposite handedness, thus favoring the *S*_4_ diastereomer (wherein four of the six ligands link metal centers of opposite configuration). Cage **10** shows the opposite behavior: the homochiral *T* diastereomer is favored by the positive *f*_2_ value (about 0.9 at room temperature). However, raising the temperature decreases the *f*_2_ value, thereby increasing the proportions of the *S*_4_ and *C*_3_ diastereomers at the expense of the *T* diastereomer. For cage **12** the *f*_2_ value was found to be close to 0 (independent of the temperature), resulting in a nearly unbiased statistical distribution (which would be 1:4:3 for *T*:*C*_3_:*S*_4_). For cage **9** some discrepancies between the data and the model were observed for the *S*_4_ and *C*_3_ diastereomers. However, its behavior can still be characterized by the moderately positive *f*_2_ value of 0.35 that slightly favors the *T* diastereomer.

**Figure 3:**
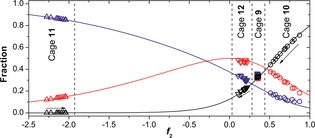
Experimental diastereomer distribution and predicted distribution as function of the *f*_2_ value (diastereomers: *T*=black; *C*_3_=red; *S*_4_=blue) for cage **9** (□), **10** (○), **11** (▵), and **12** (▿). Arrows indicate increasing temperature.

The direction and sensitivity of the change in *f*_2_ value with respect to temperature are determined by the sign and magnitude of the enthalpic contribution to the free energy. The enthalpic and entropic components of *f*_2_ can be extracted by a Van't Hoff type analysis and can be shown by our model to relate to all three of the pairwise equilibria6b between the *T*, *S*_4_, and *C*_3_ states (see Section S6 in the Supporting Information). The generally good agreement between the separately determined entropies and enthalpies and those derived from our model further validates the applicability of the model, providing a unifying understanding of the equilibria. Apart from the case of cage **9**, we found that it is entropically favorable to have opposite stereoconfiguration at the two ends of each ligand, even when that combination is enthalpically unfavorable.

Our statistical mechanical model thus accounts for the specific nonlinear response of excess chirality in metal–organic tetrahedra due to stereochemical communication within the structures. For the first time for metal–organic cages, the effect of structural features on the degree of stereochemical communication has been quantified in terms of two energy parameters with clearly defined physical meanings, and an overarching description of the temperature‐dependent equilibrium between diastereomeric cages has been provided. The general nature of the model allows for its extension to new cage geometries^19^ and related structures, such as metal–organic frameworks.^20^ We anticipate that the physical insight our model provides into the nuanced stereochemistry of the chirotopic cavities of these structures will translate into control over stereoselective guest binding and catalysis.8b, 9b

## Supporting information

As a service to our authors and readers, this journal provides supporting information supplied by the authors. Such materials are peer reviewed and may be re‐organized for online delivery, but are not copy‐edited or typeset. Technical support issues arising from supporting information (other than missing files) should be addressed to the authors.

Supplementary

## References

[1] T. R. Cook , P. J. Stang , Chem. Rev. 2015, 115, 7001. 25813093 10.1021/cr5005666

[2a] M. Yoshizawa , J. K. Klosterman , M. Fujita , Angew. Chem. Int. Ed. 2009, 48, 3418; 10.1002/anie.200805340 19391140

[2b] M. Yamashina , Y. Sei , M. Akita , M. Yoshizawa , Nat. Commun. 2014, 5, 4662; 25130933 10.1038/ncomms5662

[2c] R. Custelcean , Chem. Soc. Rev. 2014, 43, 1813; 24384869 10.1039/c3cs60371g

[2d] S. Löffler , J. Lübben , L. Krause , D. Stalke , B. Dittrich , G. H. Clever , J. Am. Chem. Soc. 2015, 137, 1060. 25569812 10.1021/ja5130379

[3] A. M. Castilla , W. J. Ramsay , J. R. Nitschke , Acc. Chem. Res. 2014, 47, 2063. 24793652 10.1021/ar5000924

[4a] B. Olenyuk , M. D. Levin , J. A. Whiteford , J. E. Shield , P. J. Stang , J. Am. Chem. Soc. 1999, 121, 10434;

[4b] J. Hamacek , D. Poggiali , S. Zebret , B. E. Aroussi , M. W. Schneider , M. Mastalerz , Chem. Commun. 2012, 48, 1281. 10.1039/c2cc17322k 22179299

[5a] Z. R. Bell , J. C. Jeffery , J. A. McCleverty , M. D. Ward , Angew. Chem. Int. Ed. 2002, 41, 2515; 10.1002/1521-3773(20020715)41:14<2515::AID-ANIE2515>3.0.CO;2-Q 12203519

[5b] O. Chepelin , J. Ujma , X. Wu , A. M. Z. Slawin , M. B. Pitak , S. J. Coles , J. Michel , A. C. Jones , P. E. Barran , P. J. Lusby , J. Am. Chem. Soc. 2012, 134, 19334; 23137068 10.1021/ja309031h

[5c] R. Annunziata , M. Benaglia , M. Cinquini , F. Cozzi , C. R. Woods , J. S. Siegel , Eur. J. Org. Chem. 2001, 173; 10.1021/jo010077l 11325283

[5d] S. E. Howson , L. E. N. Allan , N. P. Chmel , G. J. Clarkson , R. van Gorkum , P. Scott , Chem. Commun. 2009, 1727; 10.1039/b821573a 19294276

[5e] K. E. Jelfs , X. Wu , M. Schmidtmann , J. T. A. Jones , J. E. Warren , D. J. Adams , A. I. Cooper , Angew. Chem. Int. Ed. 2011, 50, 10653; 10.1002/anie.201105104 21928449

[5f] Y. Ye , T. R. Cook , S.-P. Wang , J. Wu , S. Li , P. J. Stang , J. Am. Chem. Soc. 2015, 137, 11896; 26332954 10.1021/jacs.5b07529

[5g] P. Bonakdarzadeh , F. Pan , E. Kalenius , O. Jurček , K. Rissanen , Angew. Chem. Int. Ed. 2015, 54, 14890; 10.1002/anie.201507295 26457686

[6a] R. W. Saalfrank , H. Maid , A. Scheurer , R. Puchta , W. Bauer , Eur. J. Inorg. Chem. 2010, 2903;

[6b] W. Meng , J. K. Clegg , J. D. Thoburn , J. R. Nitschke , J. Am. Chem. Soc. 2011, 133, 13652; 21790184 10.1021/ja205254s

[6c] S. P. Argent , T. Riis-Johannessen , J. C. Jeffery , L. P. Harding , M. D. Ward , Chem. Commun. 2005, 4647. 10.1039/b509239f 16175279

[7] D. Ray , J. T. Foy , R. P. Hughes , I. Aprahamian , Nat. Chem. 2012, 4, 757. 22914198 10.1038/nchem.1408

[8a] C. J. Brown , R. G. Bergman , K. N. Raymond , J. Am. Chem. Soc. 2009, 131, 17530; 19950985 10.1021/ja906386w

[8b] C. Zhao , F. D. Toste , K. N. Raymond , R. G. Bergman , J. Am. Chem. Soc. 2014, 136, 14409; 25265509 10.1021/ja508799p

[8c] Y. Nishioka , T. Yamaguchi , M. Kawano , M. Fujita , J. Am. Chem. Soc. 2008, 130, 8160. 18540605 10.1021/ja802818t

[9a] D. Fiedler , D. H. Leung , R. G. Bergman , K. N. Raymond , J. Am. Chem. Soc. 2004, 126, 3674; 15038695 10.1021/ja039225a

[9b] T. Liu , Y. Liu , W. Xuan , Y. Cui , Angew. Chem. Int. Ed. 2010, 49, 4121; 10.1002/anie.201000416 20458719

[9c] W. Xuan , M. Zhang , Y. Liu , Z. Chen , Y. Cui , J. Am. Chem. Soc. 2012, 134, 6904; 22494630 10.1021/ja212132r

[9d] L. Martínez-Rodríguez , N. A. G. Bandeira , C. Bo , A. W. Kleij , Chem. Eur. J. 2015, 21, 7144. 25807915 10.1002/chem.201500333

[10] K. Soai , T. Kawasaki , A. Matsumoto , Acc. Chem. Res. 2014, 47, 3643. 25511374 10.1021/ar5003208

[11] S. K. Jha , K. S. Cheon , M. M. Green , J. V. Selinger , J. Am. Chem. Soc. 1999, 121, 1665.

[12] C. Piguet , Chem. Commun. 2010, 46, 6209. 10.1039/c0cc00811g 20607190

[13] N. Ousaka , J. K. Clegg , J. R. Nitschke , Angew. Chem. Int. Ed. 2012, 51, 1464; 10.1002/anie.201107532 22213279

[14] J. Clayden , M. Pickworth , L. H. Jones , Chem. Commun. 2009, 547. 10.1039/b817527f 19283286

[15a] M. Borkovec , G. J. M. Koper , C. Piguet , Curr. Opin. Colloid Interface Sci. 2006, 11, 280;

[15b] G. Koper , M. Borkovec , J. Phys. Chem. B 2001, 105, 6666;

[15c] G. Ercolani , L. Schiaffino , Angew. Chem. Int. Ed. 2011, 50, 1762; 10.1002/anie.201004201 21328640

[16a] S. E. Howson , L. E. N. Allan , N. P. Chmel , G. J. Clarkson , R. J. Deeth , A. D. Faulkner , D. H. Simpson , P. Scott , Dalton Trans. 2011, 40, 10416; 21743936 10.1039/c1dt10588d

[16b] A. M. Castilla , N. Ousaka , R. A. Bilbeisi , E. Valeri , T. K. Ronson , J. R. Nitschke , J. Am. Chem. Soc. 2013, 135, 17999. 24182351 10.1021/ja410117q

[17a] N. Ousaka , S. Grunder , A. M. Castilla , A. C. Whalley , J. F. Stoddart , J. R. Nitschke , J. Am. Chem. Soc. 2012, 134, 15528; 22916838 10.1021/ja306615d

[17b] E. A. Opsitnick , X. Jiang , A. N. Hollenbeck , D. Lee , Eur. J. Org. Chem. 2012, 708.

[18] S. Ma , M. M. J. Smulders , Y. R. Hristova , J. K. Clegg , T. K. Ronson , S. Zarra , J. R. Nitschke , J. Am. Chem. Soc. 2013, 135, 5678. 23506256 10.1021/ja311882h

[19a] C. Gütz , R. Hovorka , C. Klein , Q.-Q. Jiang , C. Bannwarth , M. Engeser , C. Schmuck , W. Assenmacher , W. Mader , F. Topić , K. Rissanen , S. Grimme , A. Lützen , Angew. Chem. Int. Ed. 2014, 53, 1693; 10.1002/anie.201308651 24453210

[19b] J.-F. Ayme , J. E. Beves , D. A. Leigh , R. T. McBurney , K. Rissanen , D. Schultz , J. Am. Chem. Soc. 2012, 134, 9488. 22554153 10.1021/ja303355v

[20] M. Yoon , R. Srirambalaji , K. Kim , Chem. Rev. 2012, 112, 1196. 22084838 10.1021/cr2003147

