# Supplementary material for: Quantification of Stereochemical Communication in Metal–Organic Assemblies
Source: Angew Chem Int Ed Engl. 2016 Jun 2;55(36):10616–20. doi: 10.1002/anie.201602968 (PMC5006869; doi:10.1002/anie.201602968)
Supplement: Supplementary file 1 — Supplementary [file ANIE-55-10616-s001.pdf]

Angewandte Chemie
Eine Zeitschrift der Gesellschaft Deutscher Chemiker

Supporting Information

Quantification of Stereochemical Communication in Metal–Organic Assemblies

Ana M. Castilla, Mark A. Miller, Jonathan R. Nitschke, and Maarten M. J. Smulders**

anie_201602968_sm_miscellaneous_information.pdf

Table of Contents

1 Synthesis and characterization S2

2 Sergeant-and-Soldiers experiments S9

2.1 General experimental procedure S9

2.2 Experiments with mononuclear complexes (**1**–**3**) S11

2.3 Experiments with $Fe^{II}_4L_6$ (**2**, **6**) and $Co^{II}_4L_6$ (**5**) cages S12

2.4 Experiment with $Co^{II}_4L_4$ cage (**8**) S14

3 Statistical Mechanical modeling S16

3.1 Summary of equations S16

3.1.1 Mononuclear complex S16

3.1.2 Tetrahedral cages with ditopic ligands S17

3.1.3 Tetrahedral cages with tritopic ligands S19

3.2 Amplification curves and limiting forms S19

3.3 Renormalization S22

3.4 Fitting procedure S23

3.5 Fitting results S24

4 Sergeant-and-Soldiers experiments with $Fe^{II}_4L_4$ cage (**7a**) S25

5 Metal-ligand Length and Strength S32

6 Van 't Hoff analysis S33

7 Additional spectroscopic data S37

8 References S47

1 Synthesis and characterization

General methods: Solvents and reagents were purchased from commercial sources and used without further purification unless specified otherwise. 6,6-diformyl-3,3-bipyridine,[S1] 5,5'-(1,4-phenylene)bis-2-pyridinecarboxaldehyde,[S2] 1,3,5-tris(2'-formylpyridyl-5')benzene,[S3] mononuclear complexes **1a**[S4] and **3a**,[S5] and cages **2a**[S6] and **5a**[S5] were prepared following literature methods. Characterization data for complexes **1b**,[S4] **1d**[S7] and cage **2b**,[S4] data for the Sergeant-and-Soldiers experiments with mononuclear complexes **1a-b** and cages **2a-b**,[S4] and the Van 't Hoff data for cages **9-12**[S8] have also been previously reported. NB: The Zn^{II}-analog of **2a** could not be prepared without an anionic template.[S9]

NMR spectra were recorded at 298 K using Bruker AVIII HD 400 MHz Smart Probe, AVIII HD 500 MHz Smart Probe and DCH 500 MHz Dual Cryoprobe spectrometers. NMR signals are reported in terms of chemical shifts (δ) in ppm, multiplicity, coupling constant (J) in Hertz (Hz), relative integral and assignment, in that order; δ_H values are referenced to the CHD_2CN residual solvent signal of CD_3CN at δ = 1.94 ppm, δ_C values are referenced to the solvent signal of CD_3CN at δ = 1.32 ppm, and δ_F values are reported relative to hexafluorobenzene at –164.9 ppm. The following abbreviations are used in reporting the multiplicity for NMR resonances: s = singlet, d = doublet, t = triplet, qu = quartet, m = multiplet, dd = doublet of doublets, td = triplet of doublets and br = broad signal. Deuterated acetonitrile (CD_3CN) was purchased from Sigma-Aldrich and used without any further purification. Spectra were processed using Bruker Topspin 3.2 (licensed to the Department of Chemistry, University of Cambridge). Assignment of all ^1H and ^{13}C resonances was achieved using standard 2D NMR techniques as ^1H–^1H COSY, ^1H–^1H NOESY and/or ^1H–^{13}C HSQC.

Low resolution electrospray ionization mass spectra (ESI-MS) were obtained on a Micromass Quattro LC, infused from a Harvard Syringe Pump at a rate of 10 μL per minute. High-resolution electrospray ionization mass spectra (HRMS) were acquired using a Thermofisher LTQ Orbitrap XL hybrid ion trap mass spectrometer (capillary temperature 30 °C; tube voltage 40 V).

Elemental analyses were obtained on an Exeter Analytical CE-440 Elemental Analyzer. CD analyses were performed on an Applied Photophysics Chirascan circular dichroism spectrometer.

Synthesis of complex 1c. Into a 10 mL vial containing CH_3CN (5 mL) were added 2-formylpyridine (15.3 mg, 0.14 mmol), (*S*)-3-methyl-2-butylamine (***S*-c**, 12.5 mg, 0.14 mmol), and iron(II) triflate [Fe(OTf)$_2$] (16.9 mg, 0.047 mmol). The vial was sealed with a rubber septum and subjected to three evacuation/nitrogen fill cycles. The solution was allowed to stir for 1 day at 40 °C. The solution volume was then reduced to 1–2 mL *via* rotary evaporation and diethyl ether added until the product precipitated out of solution. Complex **1c** was isolated by centrifugation as a purple powder and dried *in vacuo* (25 mg, 60%).^{1}H NMR (400 MHz, CD_3CN) δ = 9.19 (s, 1H, *mer* imine), 9.13 (s, 1H, *mer* imine), 8.98 (s, 1H, *mer* imine), 8.95 (s, 1H, *fac* imine), 8.34 (m, 3H, overlapping, *fac*/*mer H*-pyr), 8.20-8.07 (8H, overlapping, *fac*/*mer H*-pyr), 7.98 (m, 1H, *mer H*-pyr), 7.65 (m, 2H, *mer H*-pyr), 7.60 (m, 1H, *mer H*-pyr), 7.53 (m, 1H, *mer H*-pyr), 7.48 (m, 2H, *fac H*-pyr), 6.87 (m, 2H, *fac H*-pyr), 3.94 (m, 2H, *fac* $C^{\alpha}H$ 3-methyl-2-butylamine), 3.56 (m, 1H, *mer* $C^{\alpha}H$ 3-methyl-2-butylamine), 3.40 (m, 1H, *mer* $C^{\alpha}H$ 3-methyl-2-butylamine), 3.18 (m, 1H, *mer* $C^{\alpha}H$ 3-methyl-2-butylamine), 1.49 (d, J = 6.7 MHz, 3H, *mer* $C^{\alpha}HCH_3$ 3-methyl-2-butylamine), 1.44 (d, J = 6.8 MHz, 6H, *fac* $C^{\alpha}HCH_3$ 3-methyl-2-butylamine), 1.41 (d, J = 6.8 MHz, 3H, *mer* $C^{\alpha}HCH_3$ 3-methyl-2-butylamine), 1.37 (d, J = 6.8 MHz, 3H, *mer* $C^{\alpha}HCH_3$ 3-methyl-2-butylamine), 1.21 (m, 2H, *mer* $C^{\beta}HCH_3$ 3-methyl-2-butylamine), 0.95 (m, 4H, overlapping, *fac*/*mer* $C^{\beta}HCH_3$ 3-methyl-2-butylamine), 0.73-0.63 (m, 12H, overlapping, *fac*/*mer* $C^{\beta}HCH_3$ 3-methyl-2-butylamine), 0.41 (d, J = 6.7 Hz, 3H, *mer* $C^{\beta}HCH_3$ 3-methyl-2-butylamine), 0.33 (d, J = 6.7 Hz, 3H, *mer* $C^{\beta}HCH_3$ 3-methyl-2-butylamine), 0.25-0.18 (m, 12H, overlapping *fac*/*mer* $C^{\beta}HCH_3$ 3-methyl-2-butylamine). ^{13}C NMR (126 MHz, CD_3CN) δ = 172.2, 171.6(2C), 171.3, 160.9, 160.1, 159.4, 158.8, 157.5(2C), 156.1, 155.4, 139.9, 139.6, 139.5, 139.4, 131.1, 130.9, 130.6, 130.4, 129.7, 129.6, 129.4(2C), 70.4, 69.1, 68.6, 68.4, 21.0, 20.3, 19.5, 18.7, 17.3, 17.2, 15.9, 15.7, 15.5, 15.1, 14.3, 14.2, 13.9, 13.9, 13.6; ESI-MS: *m/z* 292.1 [**1c**]$^{2+}$, 733.4 [**1c**(OTf)]$^{+}$. Elemental analysis (%) calcd for $C_{35}H_{48}F_6FeN_6O_6S_2 \cdot 2.3H_2O$: C 45.49, H 5.74, N 9.09; found C 45.04, H 5.26, N 8.74.

Synthesis of complex 3b. Into a 10 mL vial containing CH_3CN (7 mL) were added 2-formylpyridine (23.7 mg, 0.22 mmol), (*S*)-1-phenylethylamine (***S*-b**, 26.9 mg, 0.22 mmol), and cobalt(II) triflate [Co(OTf)$_2$] (26.4 mg, 0.07 mmol). The vial was sealed with a rubber septum and subjected to three evacuation/nitrogen fill cycles. The solution was allowed to stir for 1 day at 40 °C. The solution volume was then reduced to 2 mL *via* rotary evaporation and diethyl ether added until the product precipitated out of solution. Complex **3b** was isolated by centrifugation as an orange powder and dried *in vacuo* (45 mg, 62%). ^{1}H NMR (400 MHz, CD_3CN), the major set of signals observed correspond to the *fac*/*Δ* isomer: δ = 269.5 (s, 3H, imine), 85.80 (br, 3H, *H*-pyr), 70.66 (s, 3H, *H*-pyr),

52.61 (s, 3H, *H*-pyr), 16.24 (br, 3 H, *H*-pyr), 15.81 (s, 3H, *H*-phenyl), 13.90 (s, 6H, *H*-phenyl), 12.87 (s, 6H, *H*-phenyl), 1.06 (d, J = 6.0 Hz, 9H, C$^{\alpha}$H*CH$_3$* phenylethylamine), –0.56 (br, 3H, C$^{\alpha}$*H* phenylethylamine); There was also a set of signals of low intensity due to the *mer*/*Δ* isomer. ESI-MS: *m/z* 344.7 [**3b**]$^{2+}$, 838.4 [**3b**(OTf)]$^{+}$. Elemental analysis (%) calcd for $C_{44}H_{42}CoF_6N_6O_6S_2 \cdot 1.9H_2O$: C 51.7, H 4.52, N 8.22; found C 51.4, H 4.17, N 8.19.

Synthesis of complex 4a. Into a 10 mL vial containing CH_3CN (7 mL) were added 2-formylpyridine (60.1 mg, 0.56 mmol), *p*-toluidine (**a**, 60.1 mg, 0.56 mmol), and zinc(II) triflate [Zn(OTf)$_2$] (68 mg, 0.19 mmol). The solution was allowed to stir for 1 day at 50 °C. The solution volume was then reduced to 1–2 mL *via* rotary evaporation and diethyl ether added until the product precipitated out of solution. Complex **4a** was isolated by centrifugation as a yellow powder and dried *in vacuo* (123 mg, 66%). ^{1}H NMR (400 MHz, CD_3CN) δ = 8.81 (br, 3H, imine), 8.56 (br, 3H, *H*-pyr), 8.26 (br, 3H, *H*-pyr), 7.93 (br, 3H, *H*-pyr), 7.86 (br, 3H, *H*-pyr), 7.07 (br, 6H, *H*-toluidine), 6.84 (br, 6H, *H*-toluidine), 2.28 (s, 9H, C*H*$_3$ *p*-toluidine); ^{13}C NMR (100 MHz, CD_3CN) δ = 160.6, 149.2, 146.5, 143.4, 142.1, 138.9, 129.8, 129.5, 129.2, 121.5, 20.0. The lability of the $Zn^{II}L_3$ complex precluded its observation by ESI-MS. Elemental analysis (%) calcd for $C_{41}H_{36}F_6N_6O_6S_2Zn \cdot 5.3\ H_2O$: C 47.0, H 4.48, N 8.02; found C 45.87, H 3.32, N 7.61.

Synthesis of complex 4b. Into a 10 mL vial containing CH_3CN (4 mL) were added 2-formylpyridine (25.6 mg, 0.24 mmol), (*S*)-1-phenylethylamine (***S*-b**, 29 mg, 0.24 mmol), and zinc(II) triflate [Zn(OTf)$_2$] (29 mg, 0.08 mmol) and the mixture was allowed to stir for 1 day at 50 °C. The solution volume was then reduced to 1–2 mL *via* rotary evaporation and diethyl ether added until the product precipitated out of solution. Complex **4b** was isolated by centrifugation as a pale yellow powder and dried *in vacuo* (64 mg, 81%). ^{1}H NMR (400 MHz, CD_3CN), the major set of signals observed correspond to the *fac*/Δ isomer: δ = 8.21 (s, 3H, imine), 8.06 (td, J = 1.4, 7.7 MHz, 3H, *H*-pyr), 7.53 (m, 3H, *H*-pyr), 7.45 (d, J = 7.7 MHz, 3H, *H*-pyr), 7.40 (br, 3H, *H*-pyr), 7.08 (t, J = 7.4 MHz, 3H, *H*-phenyl), 6.9 (t, J = 7.8 MHz, 6H, *H*-phenyl), 6.64 (d, J = 7.9 MHz, 6H, *H*-phenyl), 5.47 (qu, J = 6.8 MHz, 3H, C$^{\alpha}$*H* phenylethylamine), 1.62 (d, J = 6.5 MHz, 9H, C$^{\alpha}$H*CH$_3$* phenylethylamine). There was also a broad set of signals of low intensity due to the *mer*/*Δ* isomer. Ratio is approximately 5:1 *fac*:*mer*; ^{13}C NMR (100 MHz, CD_3CN) *fac*/*Δ* isomer: δ = 161.7, 147.8, 146.2, 141.5, 140.5, 129.9, 129.3, 128.8, 127.7, 125.6, 64.2, 22.7. Signals corresponding to the *mer* isomer were too weak to be observed;[S10] ESI-MS: *m/z* 347.5 [**4b**]$^{2+}$; Elemental analysis (%) calcd for $C_{44}H_{42}F_6N_6O_6S_2Zn \cdot 0.75\ H_2O$: C 52.44, H 4.35, N 8.34; found C 52.64, H 4.31, N 8.10.

Synthesis of cage 2c·[OTf]$_8$: Into a 10 mL vial containing CH_3CN (2 mL) were added 6,6-diformyl-3,3-bipyridine (5.11 mg, 24 μmol), (*S*)-3-methyl-2-butylamine (***S*-c**, 4.4 mg, 50 μmol), and iron(II) triflate [Fe(OTf)$_2$] (5.7 mg, 16 μmol). The vial was sealed with a rubber septum and subjected to three evacuation/nitrogen fill cycles. The solution was sonicated for 5 min and stirred overnight at 70 °C. The reaction mixture was allowed to cool to room temperature and filtered through glass fiber. Cage **2c** was precipitated as a purple crystalline solid by addition of diethyl ether to the filtrate (yield 9 mg, 63%). ^1H NMR (400 MHz, CD_3CN): δ = 9.08 (s, 12H, imine), 8.49 (d, J = 8.2 Hz, 12H, 5,5'-bipyridine), 7.75 (d, J = 7.9 Hz, 12H, 4,4'-bipyridine), 6.46 (s, 12H, 2,2'-bipyridine), 4.04 (m, 12H, $C^{\alpha}H$ 3-methyl-2-butylamine), 1.47 (d, J = 7.2 Hz, 36H, $C^{\alpha}HCH_3$ 3-methyl-2-butylamine), 0.79 (d, J = 6.1 Hz, 36H, $C^{\beta}HCH_3$ 3-methyl-2-butylamine), 0.65 (m, 12H, $C^{\beta}H$ 3-methyl-2-butylamine), 0.27 (d, J = 6.5 Hz, 36H, $C^{\beta}HCH_3$ 3-methyl-2-butylamine); ^{13}C NMR (125 MHz, CD_3CN): δ = 172.2, 159.6, 154.2, 139.5, 136.4, 130.5, 71.4, 31.0, 20.6, 15.7, 13.7; ^{19}F NMR (376 MHz, CD_3CN): δ = –77.9 (s, *endo* OTf^-), –79.1 (bs, *exo* OTf^-) (hexafluorobenzene as an internal standard); ESI-MS: *m/z* 353.16 [**2c**(OTf)]$^{7+}$, 437.28 [**2c**(OTf)$_2$]$^{6+}$, 554.64 [**2c**(OTf)$_3$]$^{5+}$, 730.62 [**2c**(OTf)$_4$]$^{4+}$, 1023.89 [**2c**(OTf)]$^{3+}$. Elemental analysis (%) calcd for $C_{140}H_{180}F_{24}Fe_4N_{24}O_{24}S_8\cdot 12H_2O$: C 45.02, H 5.51, N 9.0; found C 44.33, H 4.81, N 8.74.

Synthesis of cage 5b·[OTf]$_8$: Into a 10 mL vial containing CH_3CN (1 mL) were added 6,6-diformyl-3,3-bipyridine (5.6 mg, 26.4 μmol), (*S*)-1-phenylethylamine (***S*-b**, 6.7 mg, 55.4 μmol), and cobalt(II) triflate [Co(OTf)$_2$] (6.3 mg, 17.6 μmol). The vial was sealed with a rubber septum and subjected to three evacuation/nitrogen fill cycles. The solution was sonicated for 5 min and stirred at 90 °C for 24h. The reaction mixture was allowed to cool to room temperature and filtered through glass fiber. Cage **5b** was precipitated as an orange solid by addition of diethyl ether to the filtrate (yield 12 mg, 70%). ^1H NMR (400 MHz, CD_3CN): δ = 274.87 (s, 12H, imine), 81.51 (s, 12H, 2-pyridine), 66.85 (s, 12H, 5-pyridine), 15.63 (br, 12H, 4-pyridine), 15.39 (s, 24H, *H*-phenyl), 14.67 (s, 12H, *H*-phenyl), 14.17 (br, 24H, *H*-phenyl). Signals corresponding to $C^{\alpha}H$ and CH_3 of phenylethylamine are expected at $\delta < -12$ ppm (outside the spectral window recorded); ^{19}F NMR (376 MHz, CD_3CN): δ = –75.9 (br, OTf^-) (hexafluorobenzene as an internal standard); ESI-MS: *m/z* 638.72 [**5b**(OTf)$_3$]$^{5+}$, 835.76 [**5b** (OTf)$_4$]$^{4+}$, 1164.02 [**5b** (OTf)$_5$]$^{3+}$. Elemental analysis (%) calcd for $C_{176}H_{156}Co_4F_{24}N_{24}O_{24}S_8\cdot 10.8\ H_2O$: C 51.12, H 4.33, N 8.13; found C 50.79, H 3.99, N 8.22.

Synthesis of cage 6a·[OTf]$_8$: Into a 10 mL vial containing CH_3CN (2 mL) were added 5,5'-(1,4-phenylene)bis-2-pyridinecarboxaldehyde (10.1 mg, 35 μmol), *p*-toluidine (**a**, 8.0 mg, 75 μmol), and iron(II) triflate [Fe(OTf)$_2$] (8.3 mg, 23.5 μmol). The vial was sealed with a rubber septum and subjected to three evacuation/nitrogen fill cycles. The solution was sonicated for 5 min and stirred overnight at 50 °C. The reaction mixture was allowed to cool to room temperature and filtered through glass fiber. Cage **6a** was precipitated as a dark blue solid by addition of diethyl ether to the filtrate (yield 18 mg, 75%). ^{1}H NMR (400 MHz, CD_3CN): δ = 8.80 (s, 12H, imine), 8.74 (dd, J = 1.9, 8.3 Hz, 12H, 4-pyridine), 8.57 (d, J = 8.2 Hz, 12H, 5-pyridine), 7.54 (s, 24H, *H*-phenyl center), 7.41 (d, J = 1.8 Hz, 12H, 2-pyridine), 7.04 (d, J = 8.3 Hz, 24H, 3-*p*-toluidine), 5.49 (d, J = 8.3 Hz, 24H, 2-*p*-toluidine), 2.34 (s, 36H, CH_3 *p*-toluidine); ^{13}C NMR (125 MHz, CD_3CN): δ = 174.4, 158.3, 153.6, 149.0, 139.9, 139.8, 137.9, 136.0, 131.2, 130.6, 128.7, 122.3, 20.9; ^{19}F NMR (376 MHz, CD_3CN): δ = –77.8 (br, OTf^-) (hexafluorobenzene as an internal standard); ESI-MS: *m/z* 553.42 [**6a**(OTf)$_2$]$^{6+}$, 693.91 [**6a**(OTf)$_3$]$^{5+}$, 904.75 [**6a**(OTf)$_4$]$^{4+}$, 1255.94 [**6a**(OTf)$_5$]$^{3+}$. Elemental analysis (%) calcd for $C_{200}H_{156}F_{24}Fe_4N_{24}O_{24}S_8\cdot 13H_2O$: C 53.92, H 4.13, N 7.55; found C 53.49, H 3.69, N 7.3.

Synthesis of cage 6b·[OTf]$_8$: Into a 10 mL vial containing CH_3CN (1 mL) were added 5,5'-(1,4-phenylene)bis-2-pyridinecarboxaldehyde (6.61 mg, 22.9 μmol), (*S*)-1-phenylethylamine (***S*-b**, 5.8 mg, 48 μmol), and iron(II) triflate [Fe(OTf)$_2$] (5.4 mg, 15.3 μmol). The vial was sealed with a rubber septum and subjected to three evacuation/nitrogen fill cycles. The solution was sonicated for 5 min and stirred at 70 °C for 24h. The reaction mixture was allowed to cool to room temperature and filtered through glass fiber. Cage **6b** was precipitated as a dark blue solid by addition of diethyl ether to the filtrate (yield 10 mg, 60%). ^{1}H NMR (400 MHz, CD_3CN): δ = 8.78 (s, 12H, imine), 8.16 (dd, J = 1.7, 8.2 Hz, 12H, 4-pyridine), 7.60 (d, J = 8.2 Hz, 12H, 5-pyridine), 7.22 (s, 24H, *H*-phenyl center), 7.06 (t, J = 7.4 Hz, 12H, 4-phenyl), 6.89 (t, J = 7.7 Hz, 24H, 3-phenyl), 6.73 (d, J = 1.3 Hz, 12H, 2-pyridine), 6.68 (d, J = 7.6 Hz, 24H, 2-phenyl), 5.38 (qu, J = 6.3 Hz, 12H, $C^{\alpha}H$ phenylethylamine), 1.98 (overlapping, $C^{\alpha}HCH_3$ phenylethylamine); ^{13}C NMR (125 MHz, CD_3CN): δ = 171.3, 158.1, 151.6, 140.6, 138.4, 136.8, 135.7, 130.0, 129.9, 128.6, 128.1, 125.7, 70.4, 26.4; ^{19}F NMR (376 MHz, CD_3CN): δ = –79.4 (s, OTf^-) (hexafluorobenzene as an internal standard); ESI-MS: *m/z* 398.82 [**6b**]$^{8+}$, 476.97 [**6b**(OTf)]$^{7+}$, 581.34 [**6b**(OTf)$_2$]$^{6+}$, 727.46 [**6b**(OTf)$_3$]$^{5+}$, 946.66 [**6b**(OTf)$_4$]$^{4+}$, 1311.72 [**3b**(OTf)$_5$]$^{3+}$. Elemental analysis (%) calcd for $C_{212}H_{180}F_{24}Fe_4N_{24}O_{24}S_8\cdot 14.8\ H_2O$: C 54.76, H 4.54, N 7.23; found C 54.38, H 4.16, N 7.09.

Synthesis of cage 7a·[OTf]$_8$: Into a 10 mL vial containing CH_3CN (4 mL) were added 1,3,5-tris(2'-formylpyridyl-5')benzene (20 mg, 50.8 μmol), *p*-toluidine (**a**, 16.9 mg, 157.6 μmol), and iron(II) triflate [Fe(OTf)$_2$] (18 mg, 50.8 μmol). The vial was sealed with a rubber septum and subjected to three evacuation/nitrogen fill cycles. The solution was sonicated for 5 min and stirred overnight at 70 °C. The reaction mixture was allowed to cool to room temperature and filtered through glass fiber. Cage **7a** was precipitated as a dark blue solid by addition of diethyl ether to the filtrate (yield 42 mg, 80%). ^{1}H NMR (500 MHz, CD_3CN) δ = 8.90 (s, 12H, imine), 8.70 (d, J = 8.2 Hz, 12H, 5-pyridine), 8.53 (d, J = 7.7 Hz, 12H, 4-pyridine), 7.31 (s, 12H, *H*-phenyl center), 7.28 (s, 12H, 2-pyridine), 7.10 (d, J = 7.9 Hz, 24H, 3-*p*-toluidine), 5.47 (d, J = 8.2 Hz, 24H, 2-*p*-toluidine), 2.38 (s, 36H, –CH_3 *p*-toluidine); ^{13}C NMR (126 MHz, CD_3CN) δ = 175.4, 158.8, 154.1, 149.0, 140.5, 139.9, 136.8, 136.1, 132.0, 130.8, 128.6, 122.3, 21.1; ^{19}F NMR (376 MHz, CD_3CN): δ = –77.7 (s, *endo* OTf$^-$), –78.3 (bs, *exo* OTf$^-$) (hexafluorobenzene as an internal standard); ESI-MS: *m/z* 662.69 [**7a**(OTf)$_3$]$^{5+}$, 865.67 [**7a**(OTf)$_4$]$^{4+}$, 1203.92 [**7a**(OTf)$_5$]$^{3+}$; Elemental analysis (%) calcd for $C_{188}H_{144}F_{24}Fe_4N_{24}O_{24}S_8\cdot 12H_2O$: C 52.83, H 3.96, N 7.86; found C 52.48, H 3.61, N 7.56.

Synthesis of cage 7b·[OTf]$_8$: Into a 10 mL vial containing CH_3CN (2 mL) were added 1,3,5-tris(2'-formylpyridyl-5')benzene (3.9 mg, 9.9 μmol), (*S*)-1-phenylethylamine (***S*-b**, 3.7 mg, 30.6 μmol), and iron(II) triflate [Fe(OTf)$_2$] (3.5 mg, 9.9 μmol). The vial was sealed with a rubber septum and subjected to three evacuation/nitrogen fill cycles. The solution was sonicated for 5 min and stirred at 90 °C for 2 days. The reaction mixture was allowed to cool to room temperature and filtered through glass fiber. Cage **7b** was precipitated as a dark purple solid by addition of diethyl ether to the filtrate (yield 6.3 mg, 60%). ^{1}H NMR (400 MHz, CD_3CN) δ = 8.86 (s, 12H, imine), 8.15 (d, J = 8.5 Hz, 12H, 4-pyridine), 7.60 (d, J = 8.4 Hz, 12H, 5-pyridine), 7.05 (t, J = 7.0 Hz, 12H, 4-phenyl), 6.94 (t, J = 7.3 Hz, 24 H, 2-phenyl), 6.77 (d, J = 7.5 Hz, 24H, 2-phenyl), 6.72 (s, 12H, *H*-phenyl center), 6.45 (s, 12H, 2-pyridine), 5.38 (m, 24H, C$^{\alpha}$*H* phenylethylamine), 2.01 (d, J = 6.0 Hz, 36H, C$^{\alpha}$HCH_3 phenylethylamine); ^{13}C NMR (126 MHz, CD_3CN) δ = 171.8, 158.3, 151.6, 140.9, 139.3, 136.7, 135.3, 130.4, 130.1, 138.3, 127.4, 125.9, 70.4, 26.4; ^{19}F NMR (376 MHz, CD_3CN): δ = −79.6 (bs, *exo* OTf$^-$), −79.8 (s, *endo* OTf$^-$), (hexafluorobenzene as an internal standard); ESI-MS: *m/z* 555.43 [**7b**(OTf)$_2$]$^{6+}$, 696.35 [**7b**(OTf)$_3$]$^{5+}$, 907.81 [**7b**(OTf)$_4$]$^{4+}$, 1259.99 [**7b**(OTf)$_5$]$^{3+}$.

Synthesis of cage 8a·[OTf]$_8$ Into a 10 mL vial containing CH_3CN (3 mL) were added 1,3,5-tris(2'-formylpyridyl-5')benzene (17.3 mg, 43.9 μmol), *p*-toluidine (**a**, 14.3 mg, 133 μmol), and cobalt(II) triflate [Co(OTf)$_2$] (15.7 mg, 43.9 μmol). The vial was sealed with a rubber septum and subjected to three evacuation/nitrogen fill cycles. The solution was sonicated for 5 min and stirred at 70 °C for 24h. The reaction mixture was allowed to cool to room temperature and filtered through glass fiber. Cage **8a** was precipitated as an orange solid by addition of diethyl ether to the filtrate (yield 27 mg, 60%).^{1}H NMR (400 MHz, CD_3CN): δ = 249.4 (s, 12H, imine), 77.4 (s, 12H, 2-pyridine), 71.7 (s, 12H, 5-pyridine), 11.1 (s, 12H, 4-pyridine), 11.0 (s, 48 H, overlapping *H*-phenyl center and –C*H*$_3$ *p*-toluidine), 9.2 (s, 24H, 3-*p*-toluidine), −11.1 (s, 24H, 2-*p*-toluidine); ^{19}F NMR (376 MHz, CD_3CN): δ = −74.1 (bs, *exo* OTf$^-$), −139.1 (s, *endo* OTf$^-$), (hexafluorobenzene as an internal standard). ESI-MS: *m/z* 529.37 [**8a**(OTf)$_2$]$^{6+}$, 665.05 [**8a**(OTf)$_3$]$^{5+}$, 868.70 [**8a**(OTf)$_4$]$^{4+}$, 1207.90 [**8a**(OTf)$_5$]$^{3+}$. Elemental analysis (%) calcd for $C_{188}H_{144}Co_4F_{24}N_{24}O_{24}S_8\cdot 12H_2O$: C 52.66, H 3.95, N 7.84; found C 52.33, H 3.68, N 7.67.

Synthesis of cage 8b·[OTf]$_8$: Trialdehyde-Co-strong amine: Into a 10 mL vial containing CH_3CN (1 mL) were added 1,3,5-tris(2'-formylpyridyl-5')benzene (8.2 mg, 20.8 μmol), (*S*)-1-phenylethylamine (***S*-b**, 7.7 mg, 63.5 μmol), and cobalt(II) triflate [Co(OTf)$_2$] (7.5 mg, 20.8 μmol). The vial was sealed with a rubber septum and subjected to three evacuation/nitrogen fill cycles. The solution was sonicated for 5 min and stirred at 70 °C for 24h. The reaction mixture was allowed to cool to room temperature and filtered through glass fiber. Cage **8b** was precipitated as an orange solid by addition of diethyl ether to the filtrate (yield 27 mg, 60%). ^{1}H NMR (400 MHz, CD_3CN): δ = 272.3 (s, 12H, imine), 79.7 (s, 12H, 2-pyridine), 69.0 (s, 12H, 5-pyridine), 14.2 (s, 12H, 4-pyridine), 12.6 (s, 12 H, *H*-phenyl center), 12.5, 10.8 and 4.33 (60H, *H*-phenyl), -0.5 (s, 12H, C$^\alpha$*H* phenylethylamine), −10.8 (s, 36H, C$^\alpha$HC*H*$_3$ phenylethylamine). ^{19}F NMR (376 MHz, CD_3CN): δ = −76.9 (bs, *exo* OTf$^-$), −137.0 (s, *endo* OTf$^-$), (hexafluorobenzene as an internal standard). ESI-MS: *m/z* 557.37 [**8b**(OTf)$_2$]$^{6+}$, 698.71 [**8b**(OTf)$_3$]$^{5+}$, 910.69 [**8b**(OTf)$_4$]$^{4+}$, 1263.88 [**8b**(OTf)$_5$]$^{3+}$. Elemental analysis (%) calcd for $C_{200}H_{168}Co_4F_{24}N_{24}O_{24}S_8\cdot 13H_2O$: C 53.69, H 4.37, N 7.51; found C 53.04, H 3.87, N 7.32.

2 Sergeant-and-Soldiers experiments

2.1 General experimental procedure

This method was previously reported for cages **2a–b** and mononuclear complexes **1a–b**.[S4] Stock solutions of the optically inactive compound (15.9 μmol for mononuclear complexes or 4 μmol for cages in 3.0 mL) (solution **I**) and the (*S*)-amine (63.8 μmol in 1.0 mL) (solution **II**) were prepared in CD_3CN. For each data point of the Sergeant-and-Soldiers experiments (Schemes S1–S3) a 400 μL aliquot of solution **I** (0.53 μmol), an x μL aliquot of **II** (0.064x μmol) and (100–x) μL of pure CD_3CN were added to a NMR tube (total solution volume = 500 μL). The tubes were sealed with rubber septa and subjected to three evacuation/nitrogen fill cycles. Unless otherwise specified, the solutions were allowed to stand for two days at 70 °C, and then for 1 day at room temperature. These solutions were used for CD measurements after 11-fold dilution with CH_3CN.

CD and UV-vis spectra recorded for each sample are presented in the following sections 2.2–2.4 along with the plots of normalized CD intensities *vs* (*S*)-amine content employed for fitting our SM model. In cases where slight precipitation was observed to occur in the NMR tube, the spectra were normalized with respect to the corresponding UV-vis spectra, to account for changes in the concentration of the sample.

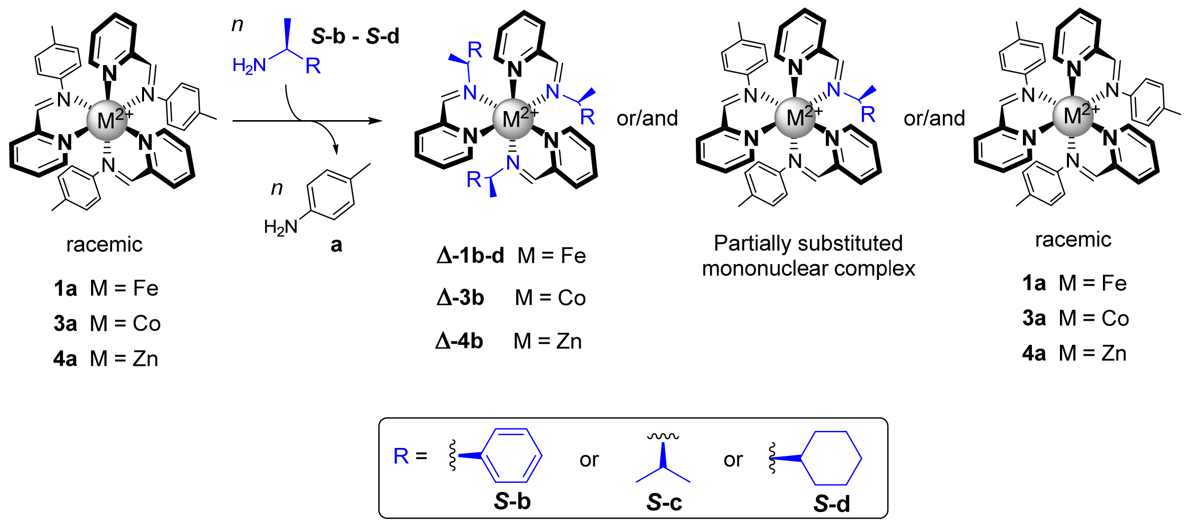

Scheme S1. Sergeant-and-Soldiers experiments with ML_3 complexes.

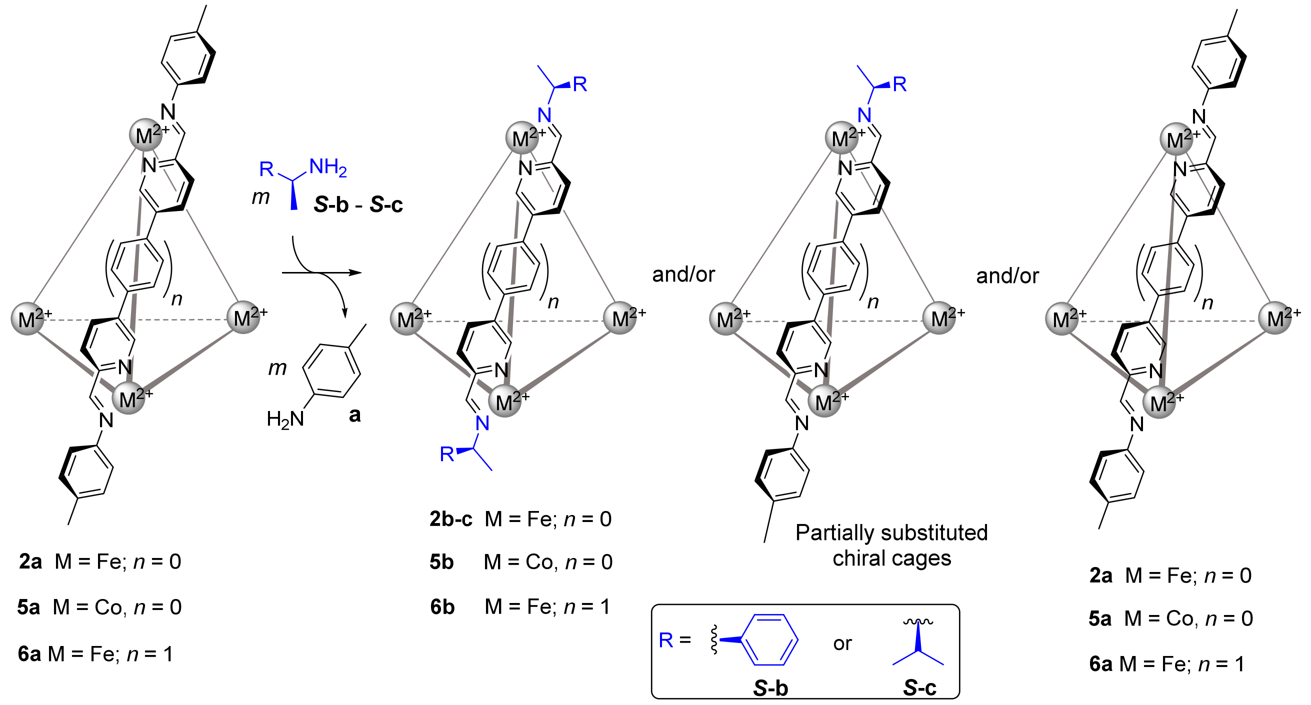

Scheme S2. Sergeant-and-Soldiers experiments with M_4L_6 cages.

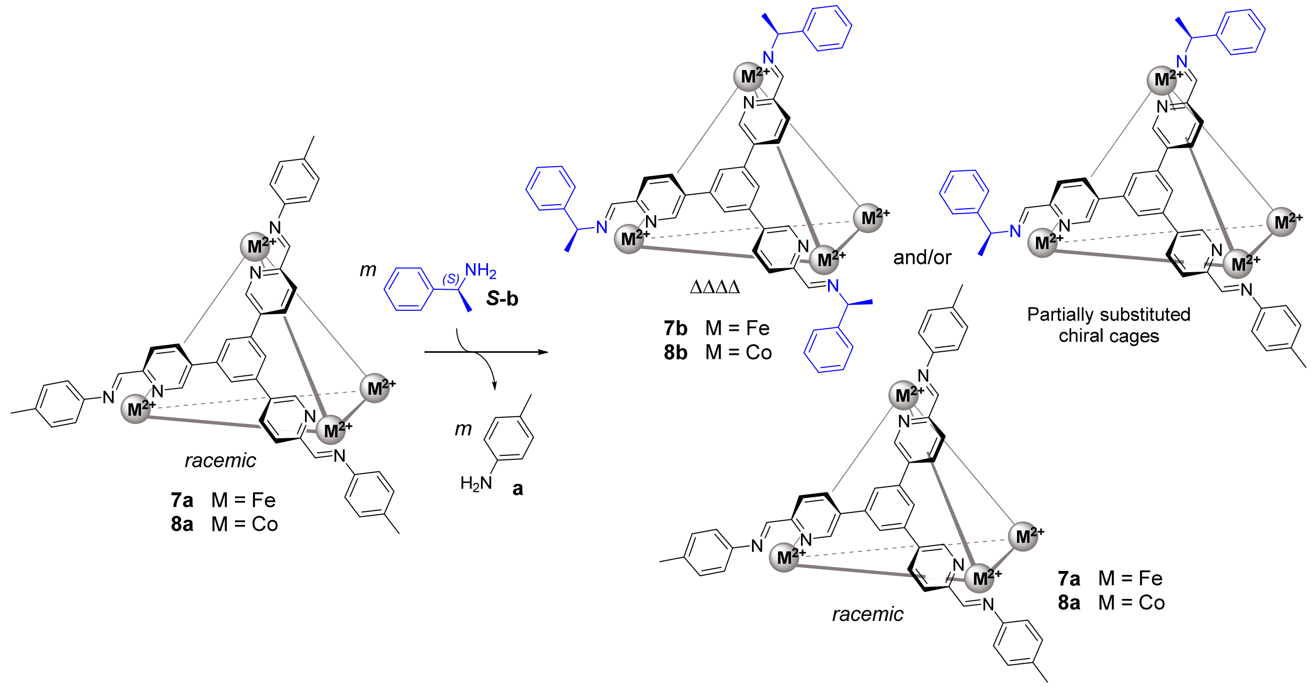

Scheme S3. Sergeant-and-Soldiers experiments with M_4L_4 cages.

2.2 *Experiments with mononuclear complexes (1–3)*

Data for the Sergeant-and-Soldiers experiment with mononuclear complex **1a** and amine ***S*-b** were previously reported.[S4]

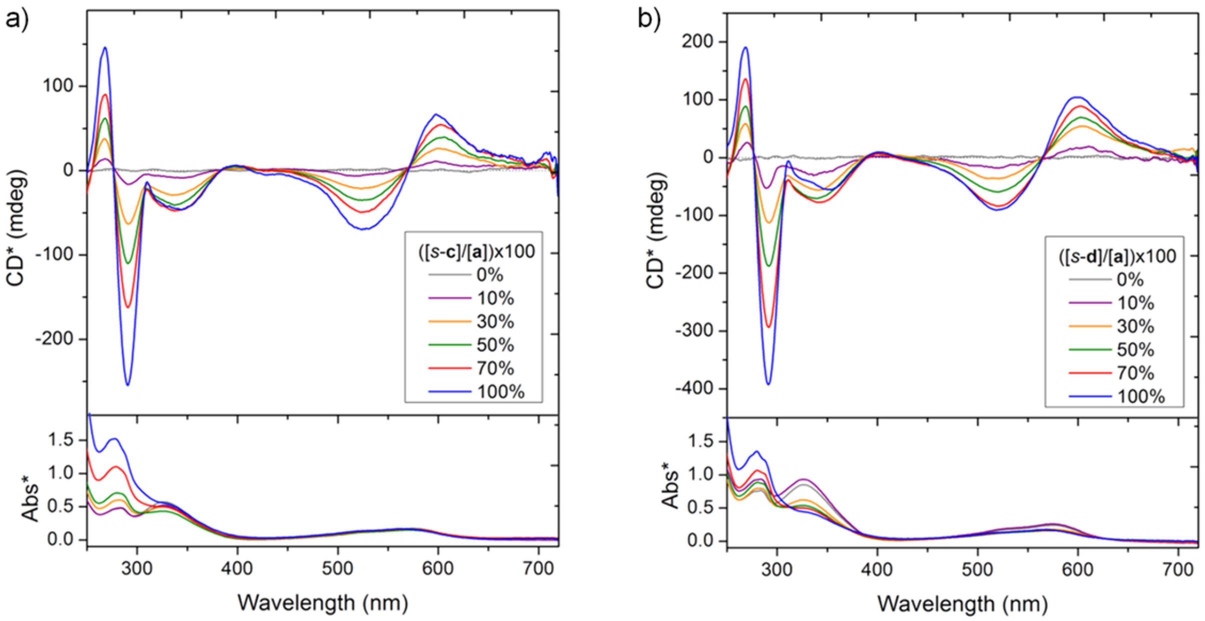

Figure S1. CD (top) and absorption (bottom) spectra of complex **1a** with different amounts of a) ***S*-c**, and b) ***S*-d** in CD_3CN/CH_3CN (1/10, v/v) at 20 °C: $[\mathbf{1a}]_0 = 3.8 \times 10^{-4}$ M, data are presented for [**a**]/[***S*-amine**] values ranging from 1/0 to 1/1. The black asterisk indicates that for each sample the spectra recorded have been normalized with respect to the corresponding absorbance intensity value at ca. 600 nm.

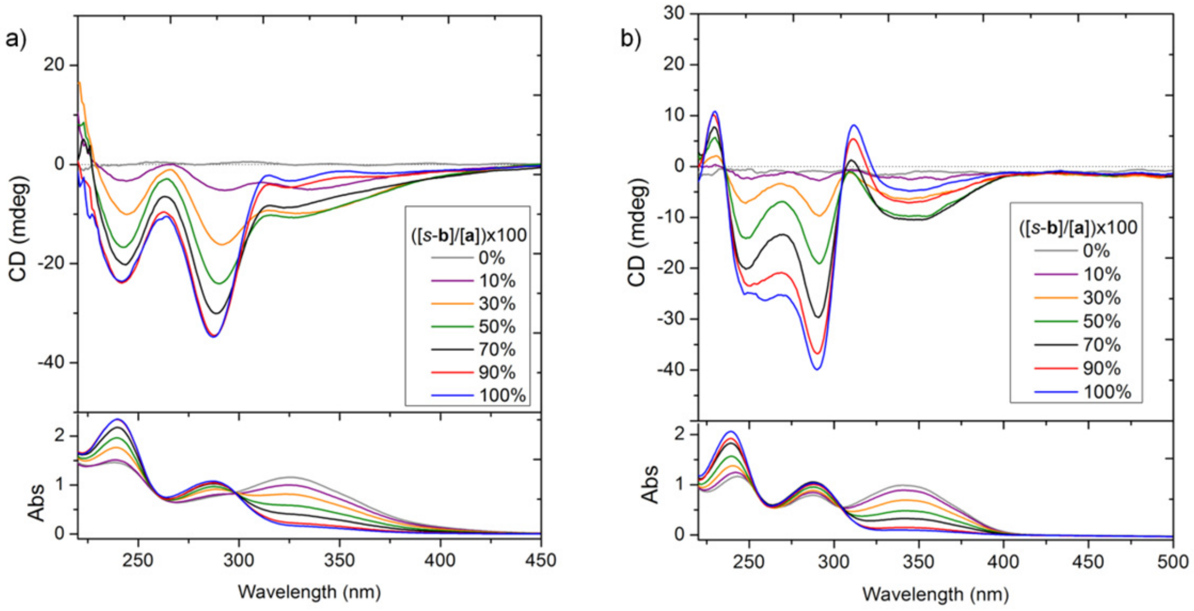

Figure S2. CD (top) and absorption (bottom) spectra of complex a) **3a** and b) **4a** with different amounts of ***S*-b**, in CD_3CN/CH_3CN (1/10, v/v) at 20 °C: $[\mathbf{3a}]_0 = [\mathbf{4a}]_0 = 3.8 \times 10^{-4}$ M, data are presented for [**a**]/[***S*-b**] values ranging from 1/0 to 1/1.

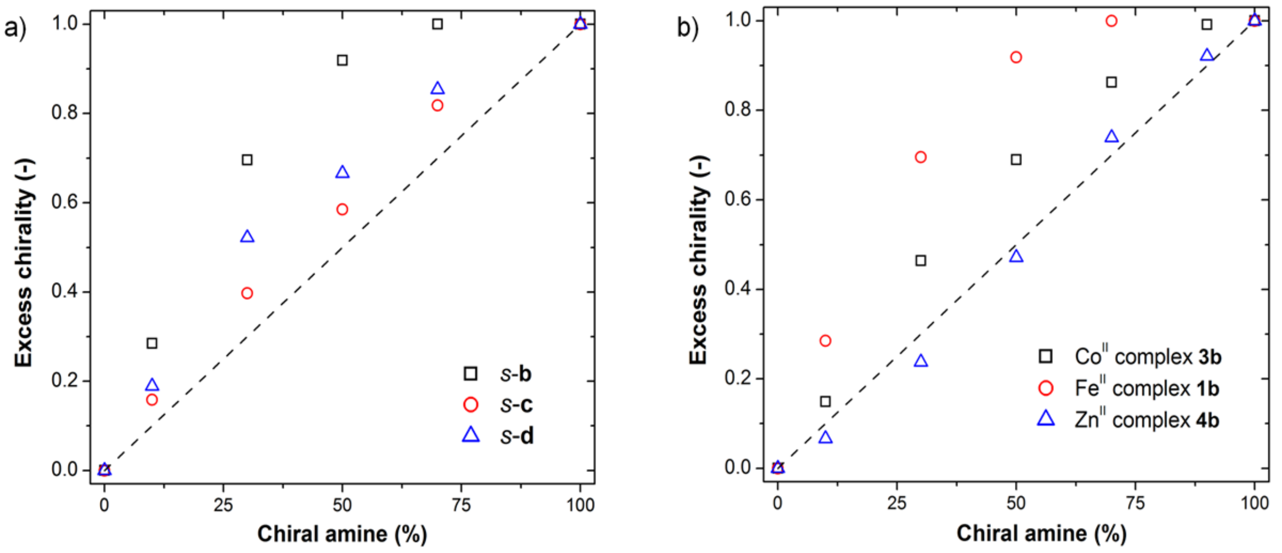

Figure S3. Comparison of the plots of normalized CD intensities (relative excess chirality) for mononuclear complexes as a function of the amount of enantiopure amine added. a) $Fe^{II}L_3$ complex (**1a**) with amines ***S*-b** – **d**. b) complexes $Fe^{II}L_3$ (**1a**), $Co^{II}L_3$ (**3a**) and $Zn^{II}L_3$ (**4a**) with amine ***S*-b**. Plotted normalized intensities at ca. 600 nm for Fe^{II} complexes and at ca. 290 nm for Co^{II} and Zn^{II} complexes. These plots are also provided in Figure 1 of the main text.

2.3 *Experiments with $Fe^{II}{}_4L_6$ (2, 6) and $Co^{II}{}_4L_6$ (5) cages*

Data for the Sergeant-and-Soldiers experiment with $Fe^{II}{}_4L_6$ cage (**2a**) and amine ***S*-b** were previously reported.[S4]

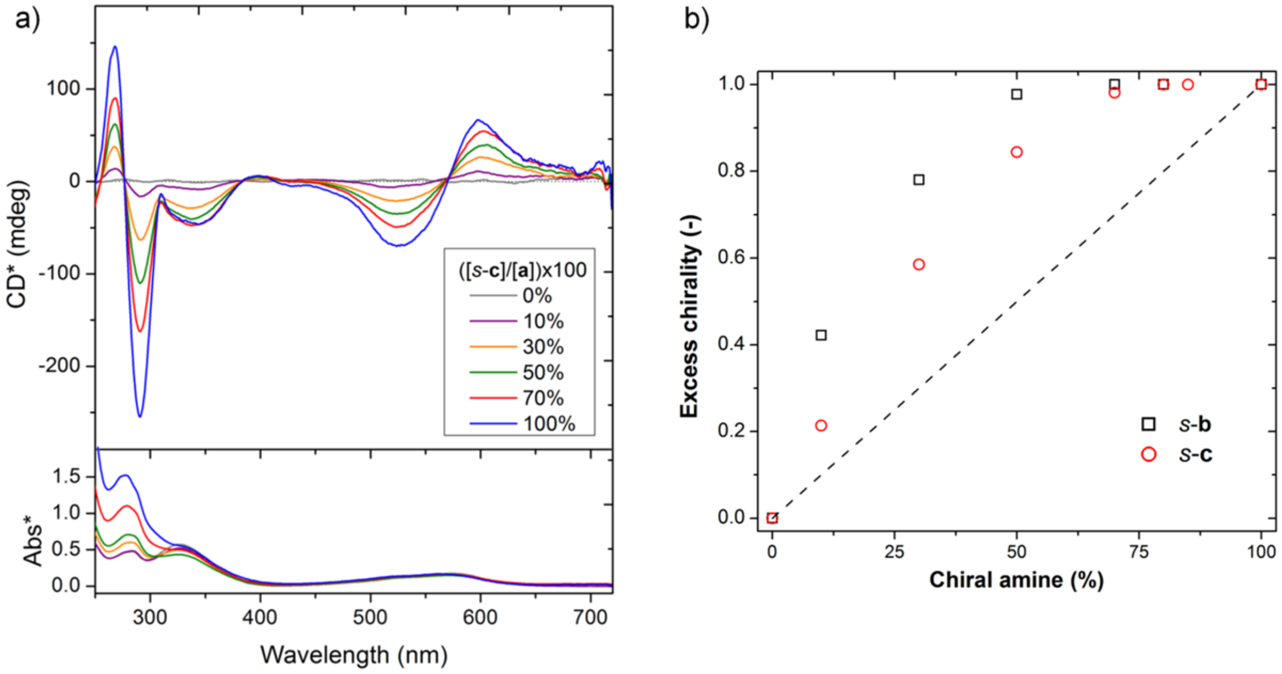

Figure S4. a) CD (top) and absorption (bottom) spectra of $Fe^{II}{}_4L_6$ cage (**2a**) with different amounts of ***S*-c**, in CD_3CN/CH_3CN (1/10, v/v) at 20 °C: $[\mathbf{2a}]_0 = 9.7 \times 10^{-5}$ M, data are presented for [**a**]/[***S*-c**] values ranging from 1/0 to 1/1. The black asterisk indicates that for each sample the spectra recorded have been normalized with

respect to the corresponding absorbance intensity value at. ca. 615 nm. b) Comparison of the plots of normalized CD intensities (relative excess chirality) at 610 nm for cage **2a** with amines ***S*-b**[S4] (black squares) and ***S*-c** (red circles) as a function of enantiopure amine content. These curves are also provided in Figure 1 of the main text.

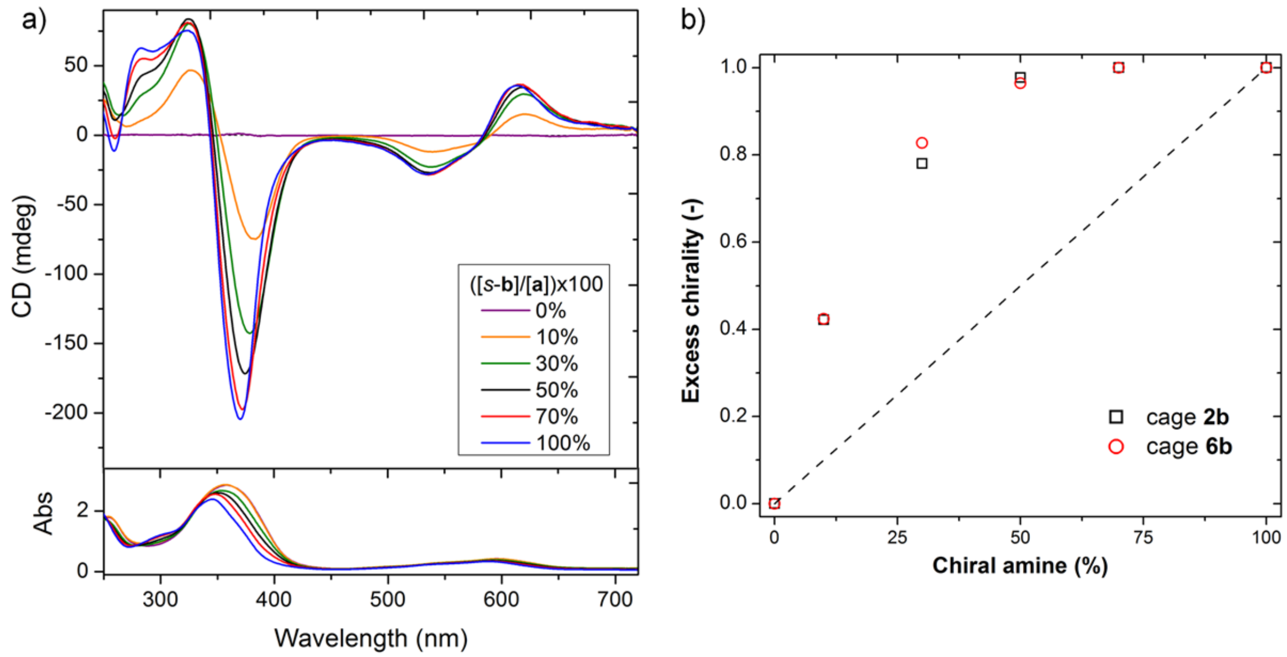

Figure S5. a) CD (top) and absorption (bottom) spectra of $Fe^{II}_4L_6$ cage (**6a**) with different amounts of ***S*-b**, in CD_3CN/CH_3CN (1/10, v/v) at 20 °C: $[\mathbf{6a}]_0 = 9.7 \times 10^{-5}$ M, data are presented for [**a**]/[***S*-b**] values ranging from 1/0 to 1/1. b) Comparison of the plots of normalized CD intensities (relative excess chirality) at 610 nm for cage **2b**[S4] (black squares) and **6b** (red circles) with ***S*-b** as a function of ***S*-b** content. These curves are also provided in Figure 1 of the main text.

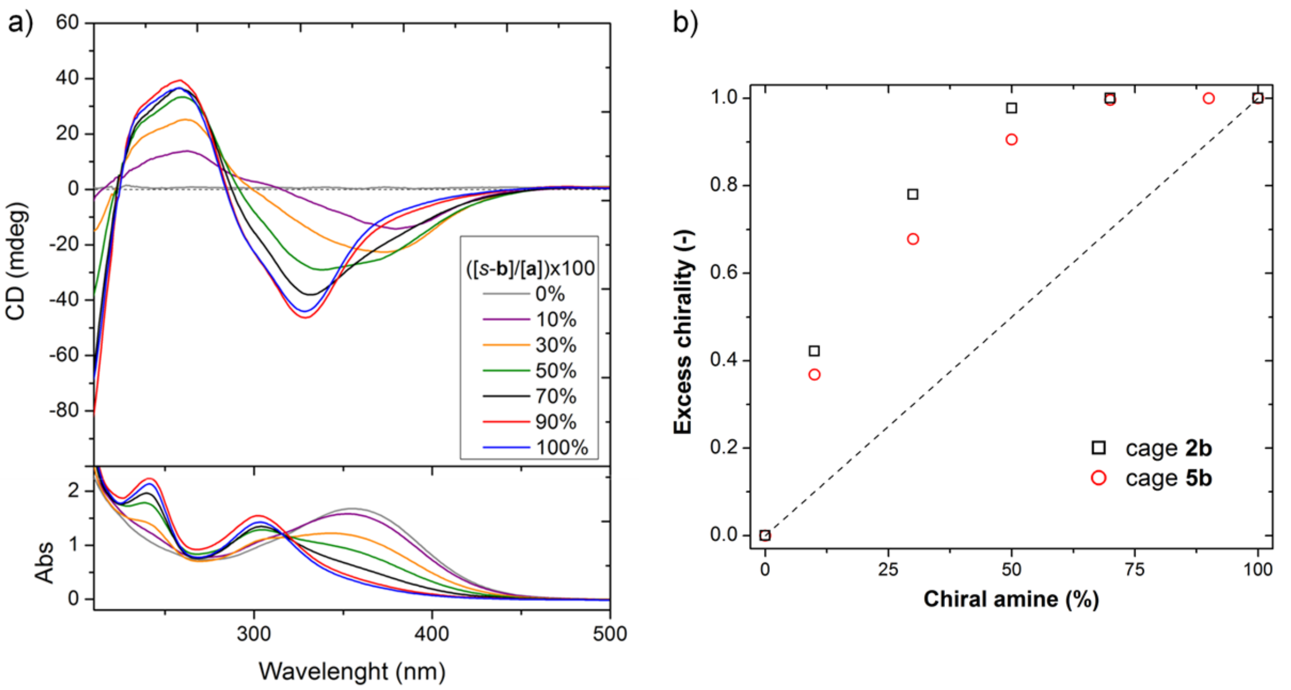

Figure S6. a) CD (top) and absorption (bottom) spectra of $Co^{II}_4L_6$ cage (**5a**) with different amounts of ***S*-b**, in CD_3CN/CH_3CN (1/10, v/v) at 20 °C: [**5a**]$_0$ = 8.6 × 10^{-5} M, data are presented for [**a**]/[***S*-b**] values ranging from 1/0 to 1/1. b) Comparison of the plots of normalized CD intensities (relative excess chirality) at 610 nm for cage **2b**[S4] (black squares) and at 258 nm for cage **5b** (red circles) with ***S*-b** as a function of ***S*-b** content. These curves are also provided in Figure 1 of the main text.

2.4 Experiment with $Co^{II}_4L_4$ cage (8)

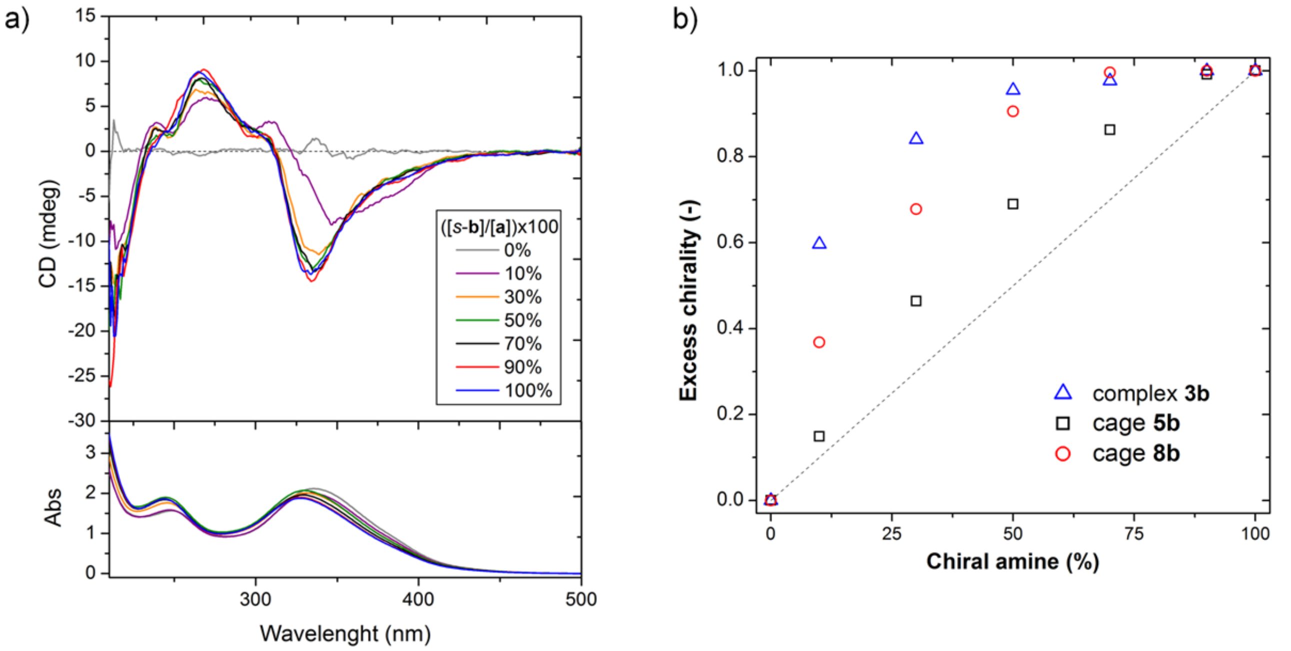

Figure S7. a) CD (top) and absorption (bottom) spectra of $Co^{II}_4L_4$ cage (**8a**) with different amounts of ***S*-b**, in CD_3CN/CH_3CN (1/10, v/v) at 20 °C: [**8a**]$_0$ = 9.8 × 10^{-5} M, data are presented for [**a**]/[***S*-b**] values ranging from 1/0 to 1/1. b) Comparison of the plots of normalized CD intensities (relative excess chirality) for Co^{II}

structures with ***S*-b** (at 290 nm for complex **3b** (black squares), at 258 nm for cage **5b** (red circles) and at ca. 340 nm for cage **8b** (blue triangles)) as a function of ***S*-b** content. These curves are also provided in Figure 1 of the main text. N.B. These mixtures were allowed to equilibrate for 12 days at 70 °C and 1 day at room temperature before dilution for CD measurements.

3 Statistical Mechanical modeling

3.1 Summary of equations

This subsection contains technical details of the statistical mechanical equations outlined in the main paper. For readers who prefer to skip this part, subsequent subsections are self-contained and include an explanation of the symbols without the equations.

The overall approach is to identify additive contributions to the free energy of a given complex and then to evaluate the Boltzmann distribution of all stereochemical states that the complex can adopt. Knowing the population of each state then allows the relative excess of Δ or Λ centers to be calculated. Our approach is closely related to a thermodynamic scheme for the entire self-assembly process of polynuclear complexes that has been reviewed by Piguet.[S11] Such models have also been devised for competitive binding of ions to polyelectrolytes.[S12] and to describe allostery, chelate and interannular cooperativity effects in supramolecular chemistry.[S13] Here, we focus exclusively on the free energy contributions relating to different stereochemical states in complexes that are already assembled. This leaves us with just two relevant parameters, allowing us to extract well-defined information from limited experimental data. The two parameters, which we will denote f_1 and f_2, are analogous to $\Delta E^{\mathrm{L,L}}$ and $\Delta E^{\mathrm{M,M}}$, respectively, in the comprehensive self-assembly model of Piguet.[S11]

3.1.1 Mononuclear complex

For a mononuclear complex ML_3 in which all three ligands L are achiral, the Δ and Λ stereochemistries have the same free energy. The Δ/Λ equivalence is removed by incorporating one or more chiral ligands. Let f_1 be the free energy, divided by the thermal energy $k_B T$, of a Λ configuration relative to Δ when one ligand is chiral. Hence, f_1 is dimensionless and is greater than zero for a ligand with a preference for Δ stereochemistry. We assume that the incorporation of two or three chiral ligands leads to an additive free energy difference. The dimensionless free energy difference between the Δ and Λ stereochemistries in an ML_3 complex in which $n = 0,1,2,3$ ligands are chiral is then nf_1. According to equilibrium Boltzmann statistics, the probabilities of a given complex with n chiral substituents adopting Δ and Λ configurations are, respectively,

$$p_n^{\Delta} = \frac{1}{1+\exp(-nf_1)} \text{ and } p_n^{\Lambda} = \frac{\exp(-nf_1)}{1+\exp(-nf_1)}.$$

In a system of independent mononuclear complexes ML_3 where a fraction *s* of the ligands are chiral, the probability of a given complex having *n* chiral ligands is

$$p_n^{\mathrm{ch}}(s) = \ {}^3C_n s^n (1-s)^{3-n},$$

where ${}^3C_n = 3!/(3-n)!\,n!$ is a binomial coefficient. The overall fraction of mononuclear complexes in a *Δ* configuration at equilibrium is the weighted sum

$$x_\Delta(s) = \sum_{n=0}^{3} p_n^{\mathrm{ch}} p_n^{\Delta} = \sum_{n=0}^{3} \frac{{}^3C_n s^n (1-s)^{3-n}}{1+\exp(-n f_1)}.$$

The fraction of *Λ* centers is $x_\Lambda(s) = 1 - x_\Delta(s)$. Hence, the fractional excess of *Δ* over *Λ* is $\chi_\Delta(s) = x_\Delta(s) - x_\Lambda(s) = 2x_\Delta(s) - 1$. For a system with equal concentrations of *Δ* and *Λ* centers, $\chi_\Delta = 0$, while for a system where all centers are *Δ*, $\chi_\Delta = 1$.

3.1.2 Tetrahedral cages with ditopic ligands

For an M_4L_6 cage, each metal center experiences a free energetic influence from any chiral substituents in the ligand directly coordinated to it, as for the mononuclear complex. In addition, a ditopic ligand may have a preference either for the same or for opposite stereochemistry at its two ends in a given structure. This results in a mutual influence (or communication of chirality) between connected metal centers. We may account for this effect by introducing a second dimensionless free energy parameter, f_2 (again, in units of k_BT), that quantifies the free energy of the *ΔΛ* and *ΛΔ* configurations of a ditopic ligand relative to the *ΔΔ* and *ΛΛ* configurations. With this convention, $f_2 > 0$ signifies a ditopic ligand with a preference for the same stereochemistry at its two ends.

Let $0 \leq n_i \leq 3$ be the number of chiral substituents coordinated to a metal center *i* ($i = 1,2,3,4$) in a M_4L_6 cage, and let m_i denote the stereochemistry of metal center *i* with $m_i = 1$ for a *Δ* center *i* and −1 for *Λ*. The dimensionless free energy of a cage with a particular combination $\{n_i\}$ of chiral substituents and metal center stereochemistries $\{m_i\}$ is

$$F(\{n_i\},\{m_i\}) = \frac{1}{2} f_1 \sum_{i=1}^{4} (1-m_i) n_i + \frac{1}{2} f_2 \sum_{i<j} (1 - m_i m_j).$$

In this equation, the first term accounts for the free energy penalty of any chiral substituents coordinated to a *Λ* center, while the second term adds the contribution from any ligands with opposite stereochemical coordination at their two ends.

The form of this free energy is that of a finite Ising model of just four "spins" (corresponding to stereochemical states in this application) and quenched disorder in the local field. In this context, the "field" is the stereochemical influence of chiral amines attached to a given metal center and the strength of the field is determined by the statistical distribution of substituted chiral amines. Once substituted at a particular location, the amines are fixed, and so the strength of stereochemical influence at a given metal does not vary, *i.e.,* it is a "quenched" variable. The fact that the number of "spins" is finite (and, indeed, very small at just 4) means that there is no true first-order phase transition or critical point, as seen for the Ising model in the thermodynamic limit. Hence, all properties change smoothly as parameters are varied.

The normalized Boltzmann weight of the set of four stereochemical configurations $\{m_i\}$ for a cage with a given combination of chiral substitutions $\{n_i\}$ is

$$p^{\mathrm{coord}}(\{n_i\},\{m_i\}) = \frac{\exp[-F(\{n_i\},\{m_i\})]}{\sum_{\{m_j\}} \exp[-F(\{n_i\},\{m_j\})]}$$

where the sum in the denominator ranges over all 16 possible combinations of stereochemical configurations at the four metal centers.

For a given set of four configurations $\{m_i\}$ in a particular complex, the fractional excess of *Δ* centers over *Λ* centers is $\chi_{\Delta}^{\mathrm{complex}}(\{m_i\}) = \frac{1}{4}\sum_{i=1}^{4} m_i$. If chiral substituents are independently distributed with probability *s*, as assumed for the mononuclear complex, then each metal center *i* has probability $p_{n_i}^{\mathrm{ch}}(s)$ of having n_i chiral substituents, identically to the mononuclear case. The overall probability of a M_4L_6 complex having a particular set $\{n_i\}$ of substitutions at its four metal centers is the product of the four individual probabilities. Hence, the statistical average of the excess of *Δ* centers over the different combinations of chiral substitutions is

$$\chi_{\Delta}(s) = \sum_{\{n_i\}} \left[\prod_{i=1}^{4} p_{n_i}^{\mathrm{ch}}(s)\right] \left[\sum_{\{m_j\}} \chi_{\Delta}^{\mathrm{complex}}(\{m_j\})\, p^{\mathrm{coord}}(\{n_i\},\{m_j\})\right]$$

where the first sum is over all possible combinations of chiral substituents at all four metal centers and the second sum is over all possible combinations of stereochemical configurations at the four centers.

3.1.3 Tetrahedral cages with tritopic ligands

A similar approach can be adopted for the tetrahedral cages M_4L_4, where L is now a tritopic ligand, as for M_4L_6 with ditopic L. The probabilities of chiral substitutions and the interpretation of the parameter f_1 are the same in the two tetrahedral complexes.

A tritopic ligand connects three metal centers that define a face of the tetrahedron, in contrast to a ditopic ligand, which connects two centers that define an edge. The three metal centers connected by a tritopic ligand can adopt either the same stereochemical configuration (*ΔΔΔ* or *ΛΛΛ*) or one center with opposite stereochemistry from the other two (*ΔΛΛ, ΛΔΔ,* and permutations). We may therefore associate a dimensionless free energy penalty f_2 with the mixed states relative to the all-*Δ* and all-*Λ* states by analogy with the ditopic case.

With this interpretation of f_2, it turns out that the contribution to the free energy of tetrahedral M_4L_6 and M_4L_4 cages with a given set of stereochemical centers is the same multiple of f_2. This can be seen by enumerating the possibilities. If all four centers have the same stereochemistry then all six edges of the tetrahedron and all four faces involve the same stereochemistry, so there is no contribution to the free energy from the parameter f_2. If one of the four metal centers has the opposite stereochemistry from the other three then there are three mixed edges (ditopic case) but also three mixed faces (tritopic). If two centers are *Δ* and the other two are *Λ* then four of the six edges are mixed (ditopic) but all four of the faces are mixed (tritopic).

Hence, all the equations for the M_4L_6 cage in the preceding section also apply to the M_4L_4 cage.

3.2 Amplification curves and limiting forms

For the mononuclear complex ML_3, the statistical mechanical (SM) model predicts the excess chirality (fraction of *Δ* centers minus that of *Λ*) as a function of the percentage of ligands that have been substituted with a chiral amine. The shape of this curve depends on the dimensionless parameter f_1, which is the free energy of a chiral amine attached to a *Λ* center relative to one attached to a *Δ* center, divided by the thermal energy $k_B T$. For a weakly directing amine, with little preference for a particular stereoconfiguration, $f_1 \approx 0$. The directing strength of a chiral amine increases with f_1 but the Sergeant-and-Soldiers effect saturates as f_1 becomes large; for a sufficiently large free energy penalty, the attachment of a single chiral amine to a metal center always results in the preferred stereochemistry, and the extent of chiral amplification only depends on the probability that at least one chiral amine is attached to a metal center.

The plots below provide example predictions of the SM model as a function of f_1.

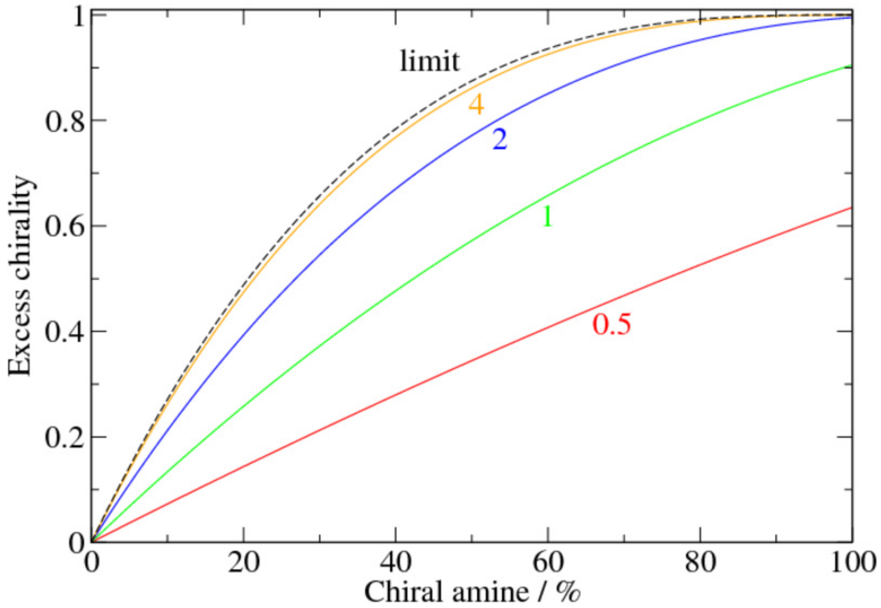

Figure S8. Prediction of the excess chirality as a function of the level of chiral amine substitution by the statistical mechanical model, for different values of the parameter f_1, as labeled on the lines. The dashed line is the limiting response for very large f_1.

These curves show that the chiral response is more sensitive to f_1 at small values, *i.e.*, when the free energetic cost of a Λ center is not large in comparison with the thermal energy $k_B T$. We also see that weakly directing amines (small f_1) produce a nearly linear response of the chiral excess to the level of substitution, while the response of strongly directing amines (large f_1) is strongly bowed.

For the tetrahedral M_4L_6 cages, the same free energetic considerations apply to each metal center. However, there may be an additional contribution to the free energy from the ditopic ligands if they have a preference for either the same stereoconfiguration at the linked metal centers ($\Delta\Delta$ or $\Lambda\Lambda$) or for different stereoconfiguration ($\Delta\Lambda$ or $\Lambda\Delta$). The parameter f_2 accounts for this tendency to transmit stereochemical information between connected centers, measuring the free energy of the heterochiral combination relative to the homochiral one, divided by $k_B T$. If $f_2 = 0$ then the ligand has no preference for either relative stereoconfiguration and there is no stereochemical communication through the ligand; the stereoconfigurations of the metal centers are independent as though they existed in mononuclear complexes. However, for $f_2 > 0$, the ligands amplify Δ stereoconfiguration by transmitting the preference for this configuration between metal centers. For example, a center with no chiral amines, and therefore no chiral preference of its own, may nevertheless be biased to Δ

stereoconfiguration if it is linked by a ligand to another center that does have a chiral substituent and that has adopted *Δ* stereochemistry.

However, it is also possible for f_2 to be negative, indicating that the heterochiral state of the ligand is more stable than the homochiral state. In this case, stereochemical communication through the ligand opposes the local bias towards *Δ* stereoconfiguration as measured by f_1 and the chiral excess is *reduced* with respect to that of the corresponding mononuclear complex.

The plots below show the effect of stereochemical transfer along ligands in the case of strongly directing amines (large f_1) and weakly directing amines (small f_1).

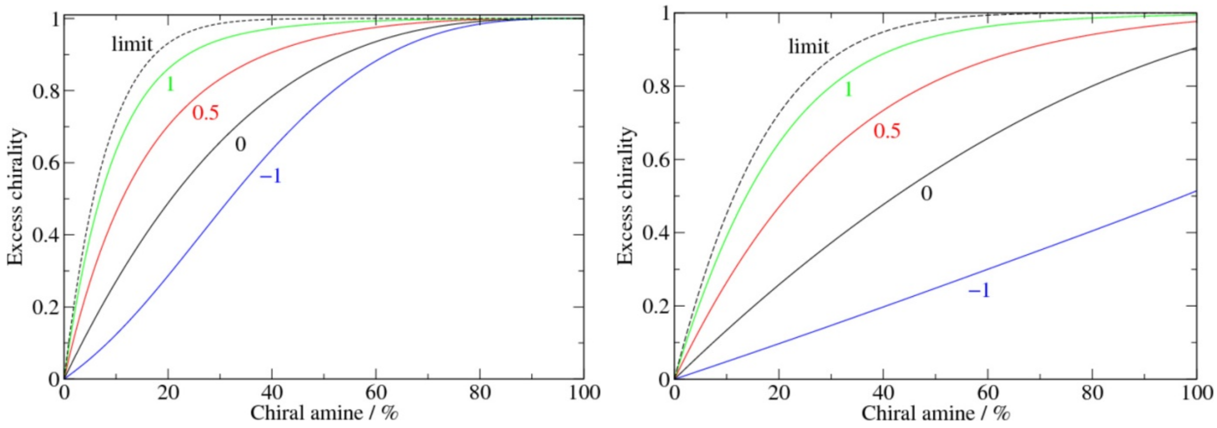

Figure S9. Influence of stereochemical coupling between centers of tetrahedral M_4L_6 cages, predicted by the SM model. The left-hand panel is for strongly directing amines (limit of large f_1) and the right-hand panel is for weakly directing amines (f_1 = 1 in this plot). In each plot, lines are labeled by the value of f_2, which measures the strength of chiral transmission. The $f_2 = 0$ lines correspond to no stereochemical communication and are equivalent to the curves for the mononuclear complex in Figure S8. Hence, the curves for $f_2 > 0$ demonstrate amplification of stereochemical information by transmission along the ligands, while the $f_2 < 0$ lines show how a ligand's preference to connect heterochiral, rather than homochiral, centers suppresses excess chirality. The limiting amplification for large positive f_2 is indicated by the dashed black lines.

For the tetrahedral M_4L_4 cages with tritopic ligands, f_2 becomes the free energy difference between the two completely homochiral configurations (*ΔΔΔ* and *ΛΛΛ*) and the remaining heterochiral configurations, which all involve one center having the opposite configuration from the other two (*ΔΛΛ, ΔΔΛ* and permutations). Once the f_2 penalties are enumerated, it turns out that the total free energy of a given combination of metal center stereoconfigurations for the M_4L_4 cage is exactly the same as the same combination for the M_4L_6 cage with ditopic ligands.

3.3 Renormalization

As Figure S8 shows, weakly directing chiral amines (small f_1) do not produce a chiral excess of unity even at 100% substitution. This is because thermal fluctuations can overcome a bias towards Δ if the bias is only weak. However, circular dichroism experiments can only measure *relative* changes in chiral excess and the absolute excess at full substitution is not known. For practical purposes, experimental excesses are therefore scaled to an excess of unity at 100% substitution. To be consistent, the SM curves must be renormalized in the same way. The plot below shows the renormalized version of Figure S8. For small f_1, this adjustment is essential for the SM model to produce the correct shapes for comparison with experiment. For large f_1, where a complete chiral excess is actually obtained at 100% substitution, the renormalization has no effect.

Once the renormalized curves have been fitted to the experimental data, it is straightforward to generate the chiral excess curves for the same parameters without renormalization. Such plots allow the absolute (rather than relative) chiral excess to be estimated. In particular the SM model provides an estimate of the maximum chiral excess at 100% substitution, a property that cannot directly be deduced from the experimental data. For example, the red line in Figure S8 shows that a weakly directing amine with $f_1 = 0.5$ leads to a maximum excess chirality of about 62%.

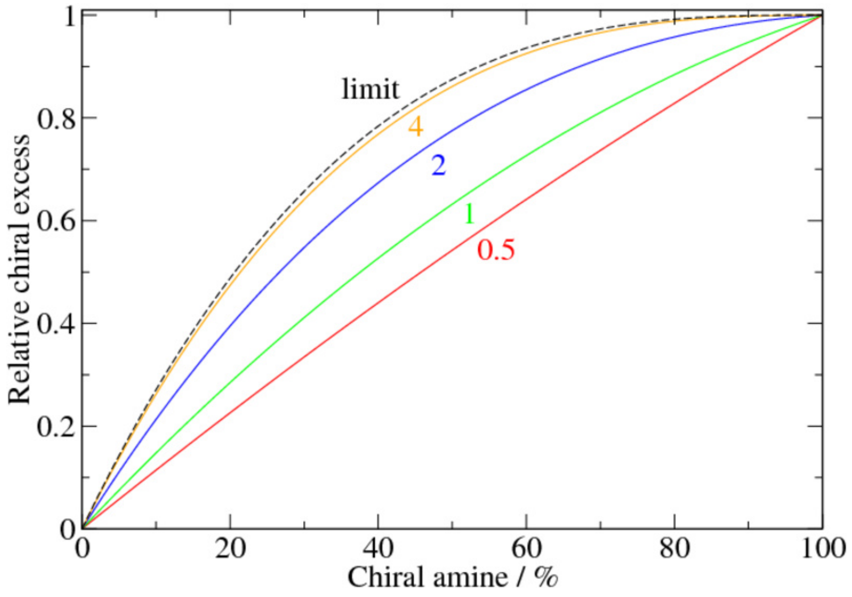

Figure S10. Predicted chiral amplification curves from the SM model for the mononuclear complex ML_3 equivalent to Figure S8, renormalized to a (relative) chiral excess of 1 at 100% chiral amine substitution.

3.4 *Fitting procedure*

All fits of the renormalized SM model to experimental data were performed using the least squares procedure implemented in the FindFit command of Mathematica 8.[S14] Values of f_1 were obtained exclusively from data from the mononuclear complexes, ML_3. These values were then transferred to corresponding cages prepared from the same metal and chiral amine, but from a ditopic and tritopic ligands. The value of f_1 was held fixed while least squares fitting was applied to obtain the parameter f_2 for stereochemical transfer along the ligands.

A free fit of f_1 and f_2 simultaneously to data for the cages resulted in unreliable fits because the unconstrained function is capable of reproducing some of the error in the experimental data if unphysical combinations of the parameters are permitted.

3.5 Fitting results

The determined energy values for all complexes is given in Table S1.

Table S1. Energy values for all studied complexes in acetonitrile.

**Complex**	**Architecture**	**Amine**	f_1	f_2
**1b**	$Fe^{II}L_3$	***S*-b**	>38	n/a
**1c**	$Fe^{II}L_3$	***S*-c**	0.90	n/a
**1d**	$Fe^{II}L_3$	***S*-d**	1.4	n/a
**2b**	$Fe^{II}{}_4L_6$	***S*-b**	>38	0.40
**2c**	$Fe^{II}{}_4L_6$	***S*-c**	0.90	0.51
**3b**	$Co^{II}L_3$	***S*-b**	1.3	n/a
**4b**	$Zn^{II}L_3$	***S*-b**	~0	n/a
**5b**	$Co^{II}{}_4L_6$	***S*-b**	1.3	0.55
**6b**	$Fe^{II}{}_4L'_6$	***S*-b**	>38	0.45
**8b**	$Co^{II}{}_4L_4$	***S*-b**	1.3	1.5

4 Sergeant-and-Soldiers experiments with $Fe^{II}_4L_4$ cage (7a)

In order to probe the degree of stereochemical communication within M_4L_4 structures we carried out a Sergeant-and-Soldiers experiment with racemic $Fe^{II}_4L_4$ cage (**7a**) and amine ***S*-b** following the general experimental procedure described in section 2.1. This substitution reaction, followed by ^{1}H NMR spectroscopy, was observed to require longer equilibration times (13 days, Figure S11) than analogous reactions with $Fe^{II}_4L_6$ cages or mononuclear complexes (2 days, see previously reported data[S4] and Figures S31–S32). The normalized CD intensities of mixtures of **7a** with different amounts of ***S*-b**, measured at ca. 610 nm, were found to vary linearly with ***S*-b** content (Figure S12), thus suggesting no stereochemical communication within the $Fe^{II}_4L_4$ framework. ESI-MS analyses of the reaction mixtures however, revealed a bimodal distribution of cages: only peaks corresponding to unsubstituted and fully (or highly) substituted cages were observed (Figure S13). This is in sharp contrast to the almost statistical distribution of all structural possibilities observed in the ESI-MS analyses of reaction mixtures of M_4L_6 cages with different amounts of ***S*-b** (see previously published experiments[S4] and Figures S33–S35). This observation confirms the previously-reported kinetic stability of the $Fe^{II}_4L_4$ framework,[S7] while it also suggests a cooperative effect in the substitution process: once the degree of substitution exceeds a critical threshold, substitution of the remaining achiral amine residues becomes more favorable. We thus attribute the observed lack of nonlinear effects in the Sergeant-and-Soldiers experiment with **7a** and ***S*-b** to the different behavior of this cage during substitution, which does not form all possible partially substituted species (in which the amplification of stereochemical information can be most strongly manifested). The cooperative effect results in the observed linear dependence of optical activity, *i.e.* the amount of enantiopure **7b** formed, with the amount of chiral amine added. Due to this non-statistical substitution behavior it was not possible to apply our SM model, which relies on the assumption that substitution of achiral amines by chiral amines results in an equal and uncorrelated probability of substitution of all amines.

Modifications of the conditions or the general methodology used for the Sergeant-and-Soldiers experiment with cage **7a** and amine ***S*-b** to favor a more rapid ligand exchange (see below) confirmed there is strong stereochemical communication between metal centers in the $Fe^{II}_4L_4$ structure.[S7] Under these modified conditions partially substituted species could be detected by ESI-MS and nonlinear effects observed during the substitution reactions. However, the data obtained did not provide quantitative information (see below).

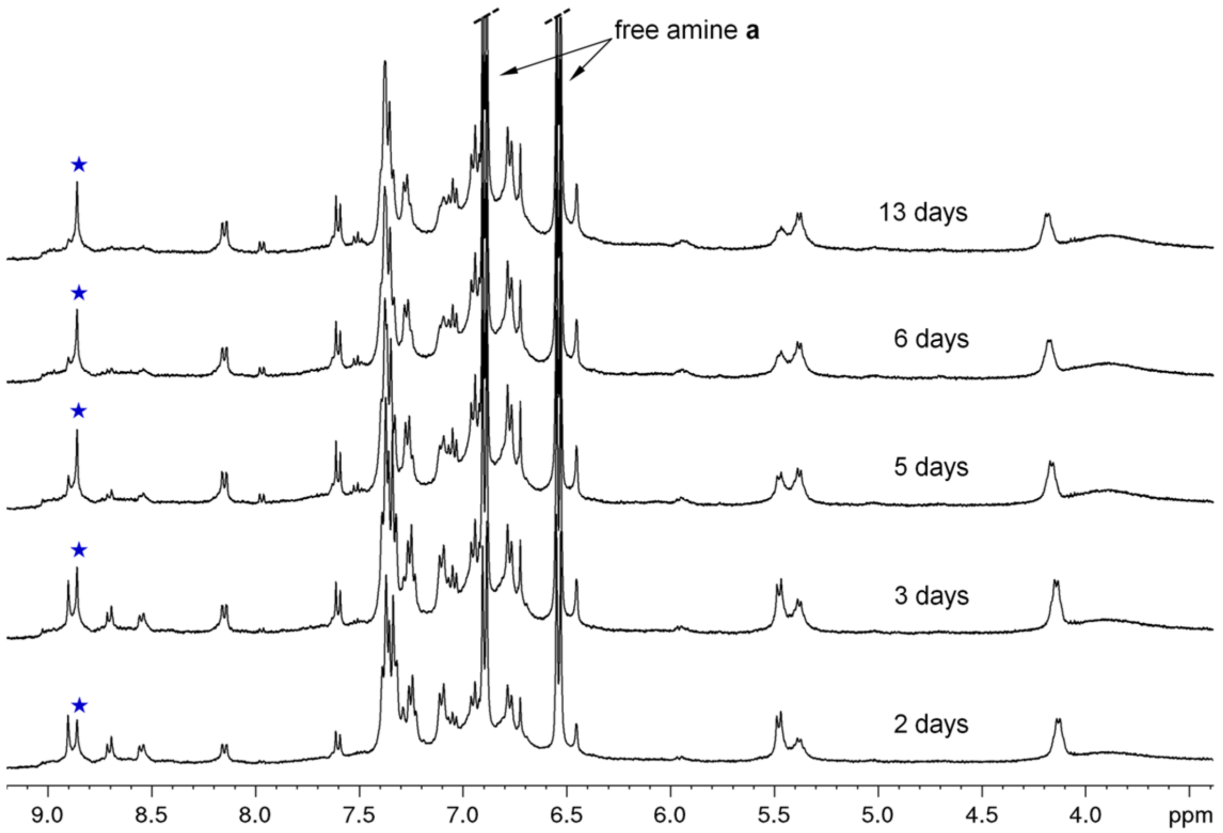

Figure S11. Partial ^{1}H NMR (400 MHz, 298 K, CD_3CN) spectra of cage **7a** during the course of its reaction with ***S*-b** in CD_3CN: $[\mathbf{7a}]_0 = 1.0 \times 10^{-3}$ M, $[\mathbf{7a}]_0/[\mathbf{\mathit{S}\text{-}b}]_0 = 1/12$. The spectra were taken after heating at 70 ºC for 2, 3, 5, 6, and 13 days. ★ denotes the imine peak of cage **7b** being formed upon reaction of **7a** with ***S*-b**.

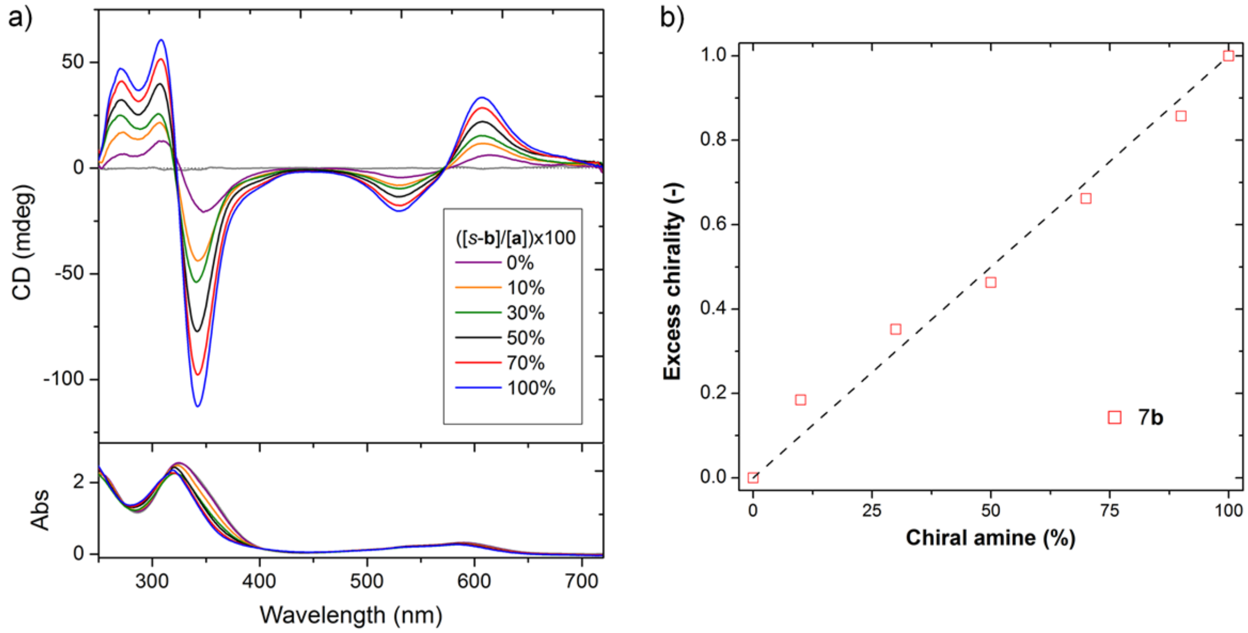

Figure S12. a) CD (top) and absorption (bottom) spectra of $Fe^{II}_4L_4$ cage (**7a**) with different amounts of ***S*-b**, in CD_3CN/CH_3CN (1/10, v/v) at 20 °C: $[\mathbf{7a}]_0 = 9.7 \times 10^{-5}$ M, data are presented for [**a**]/[***S*-b**] values ranging from 1/0 to 1/1. b) Plot of normalized CD intensities (relative excess chirality) at 610 nm for **7a** with ***S*-b** as a function of ***S*-b** content. N.B.: These mixtures were equilibrated for 13 days at 70 °C and 1 day at room temperature before dilution for CD measurements.

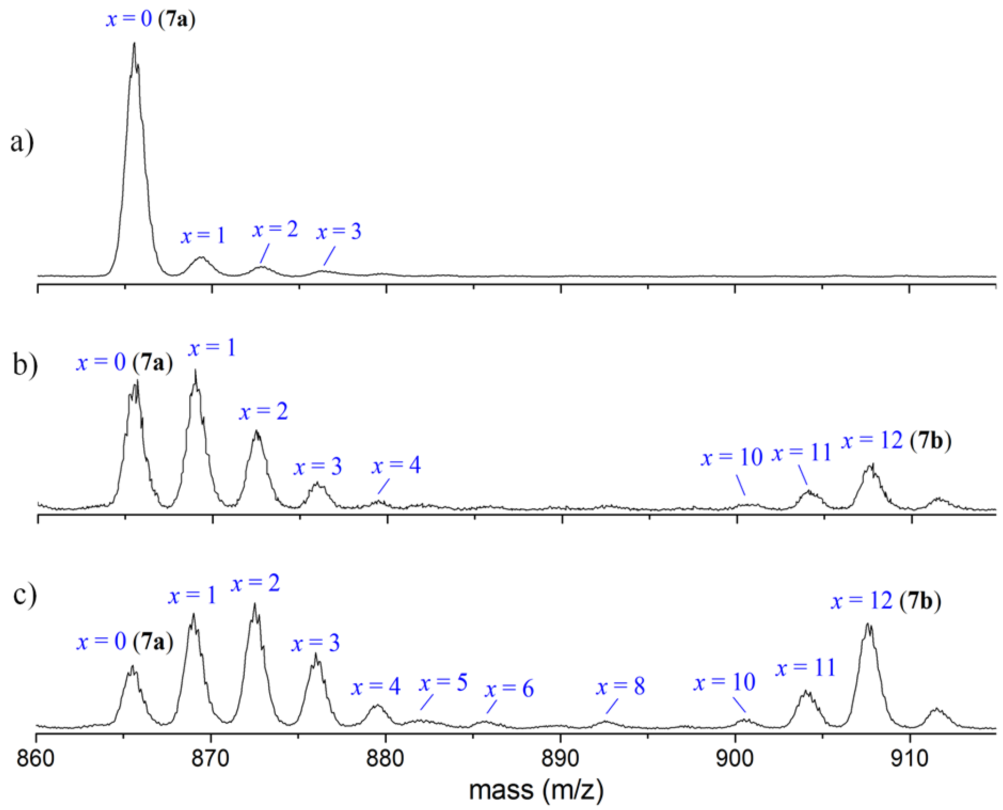

Figure S13. ESI-MS spectra (+4 peak) of **7a** with different amounts of *S*-**b** after heating for 13 days at 70 °C and 1 day at room temperature. a) 3.6 equiv of ***S*-b**. b) 6.0 equiv of ***S*-b**. c) 10.8 equiv of ***S*-b**. x = number of ***S*-b** residues incorporated in the $Fe^{II}_4L_4$ framework substituting **a** residues, where $x = 0$ corresponds to cage **7a** and $x = 12$ to **7b**.

Sergeant-and-Soldiers experiment with 7a and S-b in the presence of chloride

We reasoned that increasing the ligand exchange kinetics during the substitution reaction of cage **7a** with ***S*-b** could result in an increased substitution rate and formation of partially substituted species, thus allowing for measuring the degree of stereochemical communication within the $Fe^{II}_4L_4$ structure. It has been previously observed that chloride ions can catalyze the ligand exchange of iron(II) complexes.[S15,S16] Thus, we carried out the Sergeant-and-Soldiers experiment following the general methodology described in section 2.1 but adding 1 equivalent of Cl^- to the mixtures of **7a** and ***S*-b** (10 μL from a concentrated solution of tetrabutylammonium chloride (TBAC) in CD_3CN). The mixtures were observed to reach equilibrium after 48 h at 70 °C (in contrast to 13 days in the absence of Cl^-) (Figure S14), while also partially substituted species were detected by ESI-MS analyses (Figure S15). CD measurements however still showed a linear dependence of optical activity of **7a** and *S*-**b** mixtures with *S*-**b** content (Figure S16). We inferred that 1 equivalent of chloride was not enough to favor the formation of partially substituted species. Unfortunately, increasing the amount of Cl^- used resulted in significant precipitation of the reaction mixtures.

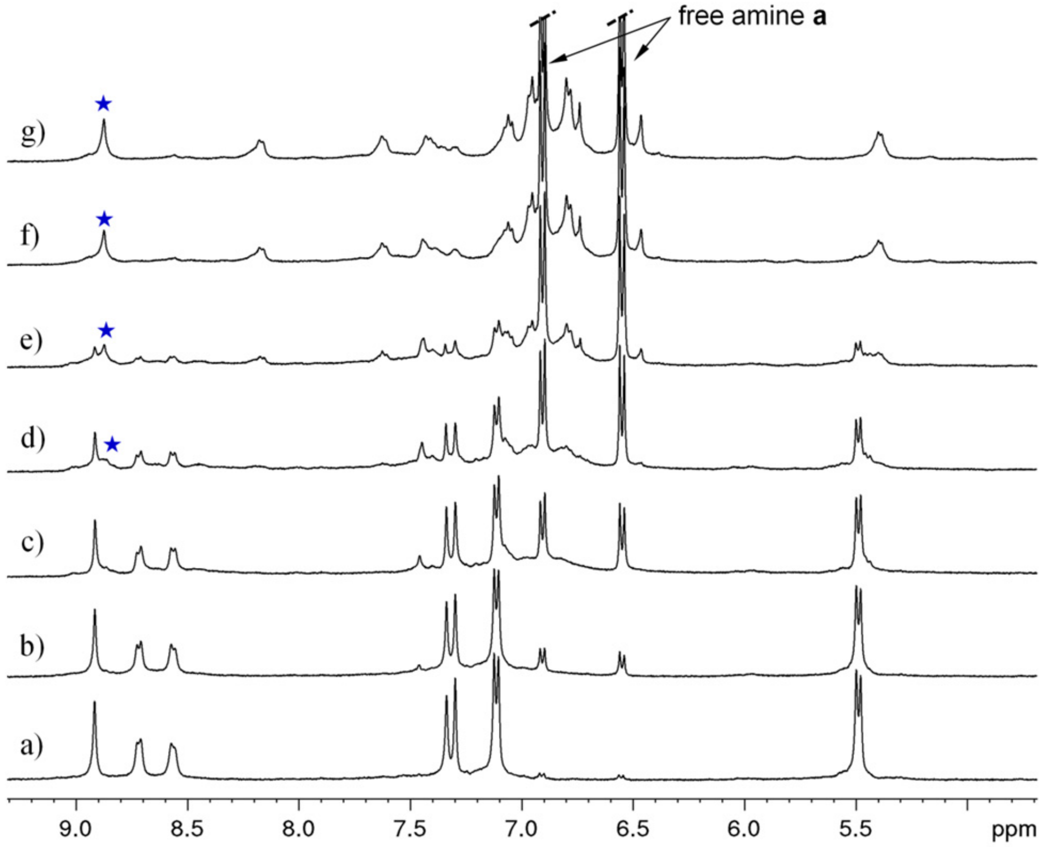

Figure S14. ^{1}H NMR (400 MHz, 298 K, CD_3CN) spectra of cage **7a** in the presence of different amount of *S*-**b** and 1 equiv of TBAC in CD_3CN: $[\mathbf{7a}]_0 = 1.3 \times 10^{-3}$ M, [*S*-**b**]/[**a**] × 100 = 0, 10, 30, 50, 70, 90 and 100%. The spectra were taken after heating at 70 °C for 2 days and then at room temperature for 1 day. Longer heating produced no further changes in the spectra.

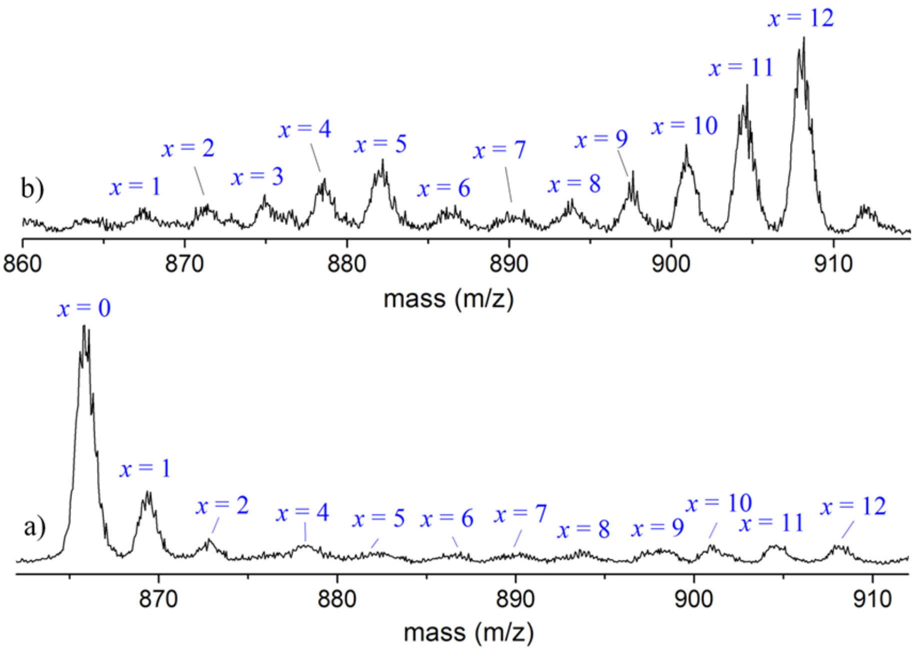

Figure S15. ESI-MS spectra (+4 peak) of **7a** with different amounts of *S*-**b** after heating for 2 days at 70 °C and 1 day at room temperature in CD_3CN and in the presence of 1 equiv of TBAC. a) 6 equiv of ***S*-b**. b) 12 equiv of ***S*-b**. x = number of ***S*-b** residues incorporated in the $Fe^{II}_4L_4$ framework substituting **a** residues, where $x = 0$ corresponds to cage **7a** and $x = 12$ to **7b**.

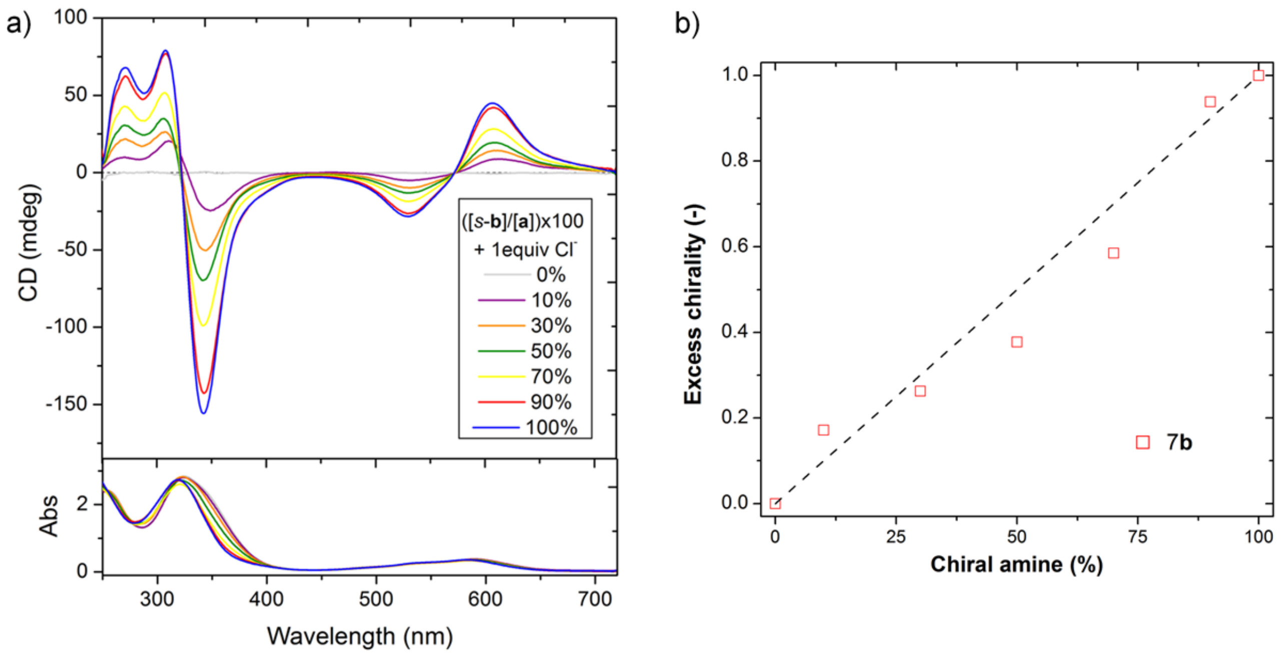

Figure S16. a) CD (top) and absorption (bottom) spectra of $Fe^{II}_4L_4$ cage (**7a**) with different amounts of ***S*-b** and in the presence of 1 equiv of TBAC in CD_3CN/CH_3CN (1/10, v/v) at 20 °C: $[\mathbf{7a}]_0 = 1.2 \times 10^{-4}$ M, data are presented for [**a**]/[***S*-b**] values ranging from 1/0 to 1/1. b) Plot of normalized CD intensities (relative excess chirality) at 610 nm for **7a** with ***S*-b** as a function of ***S*-b** content.

Modified methodology for the Sergeant-and-Soldiers experiment (through subcomponent self-assembly)

In order to favor the formation of cage **7** containing different ratios of amine **a** and ***S*-b** we carried out a modified Sergeant-and-Soldiers experiment for this cage. We prepared cage **7a** from subcomponents in the presence of different amounts of ***S*-b** instead of substituting the preformed cage **7a** with *S*-**b** (see Scheme S4 and the experimental procedure below). ESI-MS analysis of the samples after equilibration for 2 days at 70 °C and 1 day at room temperature confirmed the formation of all structural possibilities: cage **7a** incorporating from 0 to 12 residues of ***S*-b** in its structure (Figure S17), while CD measurements showed a nonlinear increase of optical activity of cage **7a** with increasing *S*-**b** content (Figure S18). This experiment thus confirmed there is stereochemical communication within the $Fe^{II}_4L_4$ framework. However, given that this experimental method used differs from the one used for the rest of the cages, these results were not included in our analysis.

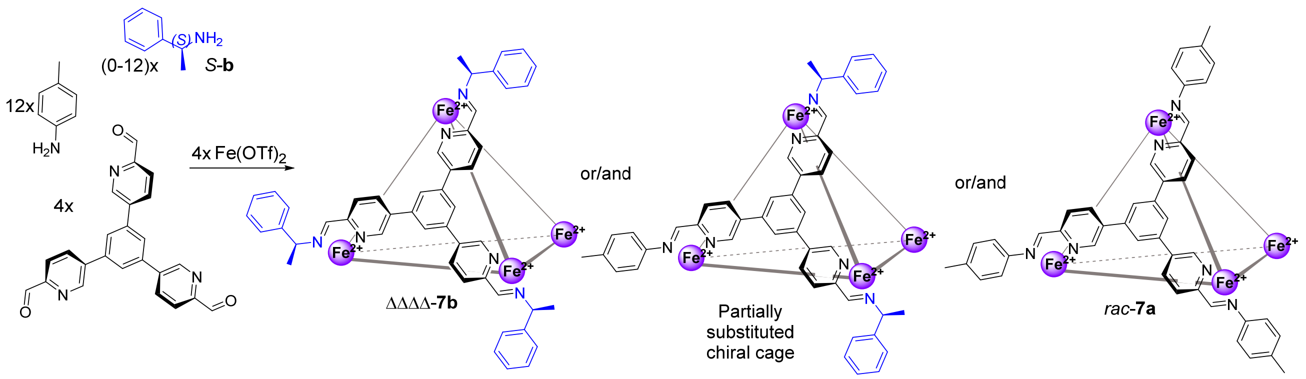

Scheme S4. Modified Sergeant-and-Soldiers experiment for $Fe^{II}_4L_4$ cage **7a** and *S*-**b**.

Method: Stock solutions of *p*-toluidine (0.2 M) (solution **I**) and *S*-**b** (0.2 M) (solution **II**) were prepared in CD_3CN. For each of the data points 1,3,5-tris(2'-formylpyridyl-5')benzene (1 mg, 2.5 μmol) and [$Fe(OTf)_2$] (0.9 mg, 2.5 μmol) were loaded into a NMR tube. A 38 μL aliquot of solution **I** (7.5 μmol), an x μL aliquot of **II** (0.2x μmol) and (438–x) μL of CD_3CN were then added to the NMR tube. The tubes were sealed with rubber septa and subjected to three evacuation/nitrogen fill cycles. The solutions were sonicated for 5 min and allowed to stand for two days at 70 °C (^{1}H NMR confirmed cages had formed and the mixtures had reached equilibrium), and then for 1 day at room temperature. These solutions were used for CD measurements after 15-fold dilution with CH_3CN.

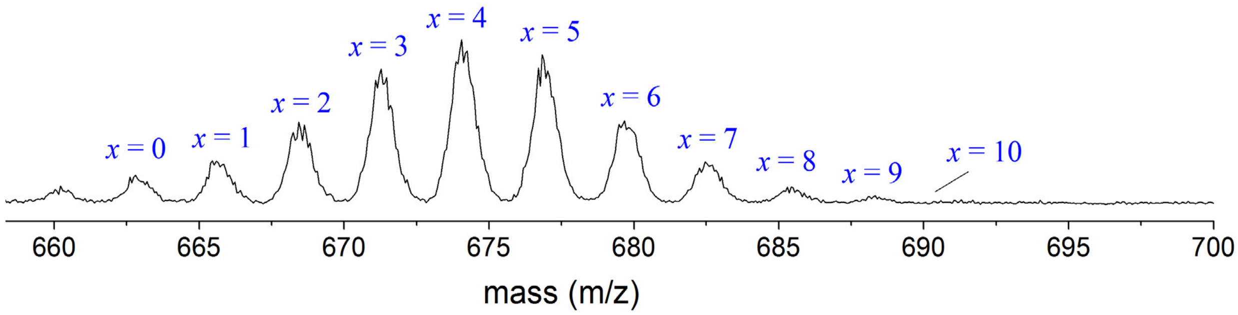

Figure S17. ESI-MS spectrum (+3 peak) of the reaction mixture of 1,3,5-tris(2'-formylpyridyl-5')benzene (4 equiv), $Fe(OTf)_2$ (4 equiv), **a** (12 equiv) and *S*-**b** (6 equiv) after heating for 2 days at 70 °C and 1 day at room temperature in CD_3CN. *x* = number of ***S*-b** residues incorporated in the $Fe^{II}_4L_4$ framework substituting **a** residues, where *x* = 0 corresponds to cage **7a** and *x* = 12 to **7b**.

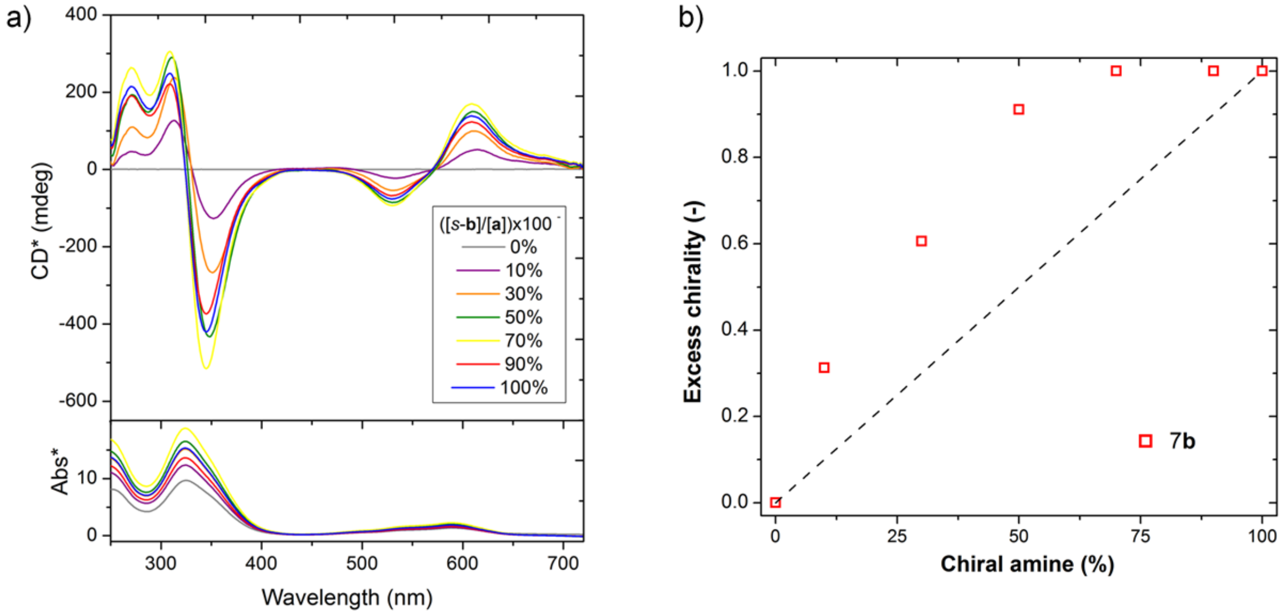

Figure S18. a) CD (top) and absorption (bottom) spectra (measured at 20 °C) of $Fe^{II}_4L_4$ cage **7a** prepared from subcomponents in the presence of different amounts of *S*-**b** in CD_3CN/CH_3CN (1/14, v/v). Data are presented for [**a**]/[*S*-**b**] values ranging from 1/0 to 1/1. b) Plot of normalized CD intensities (relative excess chirality) at 610 nm for **7a** with *S*-**b** as a function of *S*-**b** content.

5 Metal-ligand Length and Strength

Table S2. Average metal-to-ligand distance for metal tris(pyridylimine) complexes.

**Metal**	**<M–N> distance (Å)[a]**
Fe^{2+}	1.971 ± 0.008
Co^{2+}	2.149 ± 0.016
Zn^{2+}	2.172 ± 0.019

[a]: data taken from Howson *et al.*[S10]

Table S3. Average metal-to-ligand distance for metal tris(bipyridine) complexes, and the corresponding stability constant.

**Metal**	**<M–N> distance (Å)**	**Reference**	**Stability constant log β_{aq} (ML_3^{2+})[a]**
Fe^{2+}	1.965 ± 0.010	Batten *et al.*[S17]	17.45
Co^{2+}	2.162 ± 0.010	Huang *et al.*[S18]	16.05
Zn^{2+}	2.151 ± 0.015	Eom *et al.*[S19]	13.74

[a]: data taken from Makrlík *et al.*[S19]

6 Van ’t Hoff analysis

For M_4L_6 cages with no chiral amine substituents, there is no preference for Δ or for Λ stereochemistry at a given center. In the statistical mechanical model, this ambivalence corresponds to setting $f_1 = 0$. However, stereochemical effects may still be transmitted between metal centers by the ligands due to the free energy penalty f_2 that is incurred for each ditopic ligand with heterochiral coordination. There are $2^4 = 16$ combinations to describe the possible stereochemistries for the four centers in a tetrahedral cage, and these can be grouped into three symmetries:

Table S4. Possible stereoconfiguration of the four metal centers in a M_4L_6 cage and corresponding symmetry, free energy penalty and relative statistical weight.

Stereoconfiguration of centers	Symmetry	Free energy penalty	Relative statistical weight
ΔΔΔΔ, ΛΛΛΛ	T	0	2
ΔΛΛΛ, ΛΔΔΔ<br>ΛΔΛΛ, ΔΛΔΔ<br>ΛΛΔΛ, ΔΔΛΔ<br>ΛΛΛΔ, ΔΔΔΛ	C_3	$3f_2$	$8e^{-3f_2}$
ΔΔΛΛ, ΛΛΔΔ<br>ΔΛΔΛ, ΛΔΛΔ<br>ΔΛΛΔ, ΛΔΔΛ	S_4	$4f_2$	$6e^{-4f_2}$

When normalized, the relative statistical weights in the final column (Table S4) above give the equilibrium populations of the three symmetries as a function of f_2 as shown by the continuous lines in Figure 2 of the main article. For example, in the “unbiased” case of no stereochemical communication along ligands ($f_2 = 0$), the statistical weights in the final column of the table give a ratio of T:C_3:S_4 symmetries of 1:4:3.

Within the assumptions of the SM model, the populations of the isomers are universal in the sense that, at a given temperature, any M_4L_6 cage should have proportions of isomers that fall on the curves at some value of f_2. We note that f_2 is a free energy divided by k_BT, and that it therefore depends on temperature. Hence, the change in population of the isomers with temperature is reflected by a change in f_2. The change reveals useful thermodynamic information about the equilibria between the isomers.

Three pairwise equilibria may be defined:[S8]

Table S5. Three pairwise equilibria between the *T*, C_3 and S_4 diastereomer of a M_4L_6 cage, with corresponding equilibrium constants.

Equilibrium	Equilibrium constant	Statistical model
$T \rightleftharpoons C_3$	$K_1 = [C_3]/[T]$	$K_1 = 4e^{-3f_2}$
$S_4 \rightleftharpoons T$	$K_2 = [T]/[S_4]$	$K_2 = \frac{1}{3}e^{4f_2}$
$C_3 \rightleftharpoons S_4$	$K_3 = [S_4]/[C_3]$	$K_3 = \frac{3}{4}e^{-f_2}$

A Van 't Hoff plot of $\ln K$ against $1/T$ can be used to obtain the enthalpic and entropic contributions to the standard Gibbs energy change for each equilibrium.[S8] However, f_2 is also a Gibbs energy (per molecule and divided by $k_\mathrm{B}T$). Hence, we may write

$$f_2 = \frac{\hbar}{k_\mathrm{B}T} - \frac{s}{k_\mathrm{B}}$$

where *h* and *s* are the enthalpic and entropic contributions, respectively, to f_2, and the lower-case letters remind us that these quantities are molecular rather than molar. A Van 't Hoff-type plot of f_2 against $1/T$ leads to values for *h* and *s*. These contributions are related to those for all three pairwise equilibria. For example, for the first equilibrium, with standard Gibbs energy change $\Delta_\mathrm{r}g° = \hbar_1 - Ts_1$,

$$\ln K_1 = -\frac{\hbar_1}{k_\mathrm{B}T} + \frac{s_1}{k_\mathrm{B}} = \ln 4 - 3\left(\frac{\hbar}{k_\mathrm{B}T} - \frac{s}{k_\mathrm{B}}\right)$$

Hence, $\hbar_1 = 3\hbar$ and $s_1 = k_\mathrm{B}\ln 4 + 3s$. We see that there is a contribution of $k_\mathrm{B}\ln 4$ to the entropy that comes from the difference in number of metal center permutations for complexes with C_3 and *T* symmetries. Similarly for the remaining two equilibria,

$$\ln k_2 = -\frac{\hbar_2}{k_\mathrm{B}T} + \frac{s_2}{k_\mathrm{B}} = \ln\frac{1}{3} + 4\left(\frac{\hbar}{k_\mathrm{B}T} - \frac{s}{k_\mathrm{B}}\right)$$

giving $\hbar_2 = -4\hbar$ and $s_2 = -k_\mathrm{B}\ln 3 - 4s$, and

$$\ln K_3 = -\frac{\hbar_3}{k_\mathrm{B}T} + \frac{s_3}{k_\mathrm{B}} = \ln\frac{3}{4} - \left(\frac{\hbar}{k_\mathrm{B}T} - \frac{s}{k_\mathrm{B}}\right)$$

giving $h_3 = -4h$ and $s_3 = k_B \ln\frac{3}{4} + s$.

These formulae explicitly show how the equilibria are related within the unifying framework of the SM model. In principle, therefore, a single Van 't Hoff analysis of f_2 for a given cage allows the enthalpy and entropy for all three pairwise equilibria to be predicted self consistently.

Using data tabulated in the supplemental material of Meng *et al.*,[S8] the following entropic and enthalpic contributions (converted to molar units) were obtained for the cages in Figure 2 of the main article.

Table S6. Entropic and enthalpic contributions (converted to molar units) obtained for the cages **9–12**, using the SM model

**Cage**	**Number of equivalent cage in Meng *et al.*[a]**	**Table number from Meng *et al.* for population data[a]**	$h/\mathrm{kJ\ mol^{-1}}$	$s/\mathrm{J\ K^{-1}\ mol^{-1}}$
**9**	**1**	S47	0.480	–1.42
**10**	**5**	S49	5.83	12.8
**11**	**6**	S51	–4.30	4.18
**12**	**7**	S74	1.69	3.82

[a]: data taken from Meng *et al.*[S8]

These values of *h* and *s* can now be used in the equations for the enthalpic and entropic contributions to each of the three pairwise equilibria of the three isomers. These predictions of the SM model are tabulated below, along with the results of values obtained from conventional Van 't Hoff analysis in the supplemental material of Meng *et al.*[S8] In all cases, enthalpies are given in kJ $\mathrm{mol^{-1}}$ and entropies in J $\mathrm{K^{-1}}$ $\mathrm{mol^{-1}}$. Uncertainties in the fits are 0.5–2 kJ $\mathrm{mol^{-1}}$ for enthalpies and 2–5 J $\mathrm{K^{-1}}$ $\mathrm{mol^{-1}}$ for entropies. No *T* isomer is observed for cage **11**, so only equilibrium 3 can be analyzed in that case.

Table S7. Comparison of the enthalpic and entropic contributions to each of the three pairwise equilibria of the three isomers as determined using our SM model and as reported previously.[S8]

cage	Eqm 1: $T \rightleftharpoons C$				Eqm 2: $S_4 \rightleftharpoons T$				Eqm 3: $C_3 \rightleftharpoons S$			
	Model		Van ’t Hoff		Model		Van ’t Hoff		Model		Van ’t Hoff	
	h_1	s_1	h_1	s_1	h_2	s_2	h_2	s_2	h_3	s_3	h_3	s_3
**9**	1.4	7.3	1.0	2.9	–1.9	–3.5	–1.6	–5.5	0.48	–3.8	0.59	2.6
**10**	18	50	19	55	–23	–60	–17	–43	5.8	10	–1.7	–12
**11**	N.A.	N.A.	N.A.	N.A.	N.A.	N.A.	N.A.	N.A.	–4.3	1.8	–4.3	1.9
**12**	5.1	23	3.6	18	–6.8	–24	–6.8	–25	1.7	1.4	3.3	6.8

The SM model and individual Van ’t Hoff fits generally match well apart from the problematic cage **9** (see Figure 2 of the main paper). Agreement for the third equilibrium of cage **10** is also poor, although we note that the absolute values of the enthalpy and entropy changes for that equilibrium are small in comparison with those for the other two equilibria in this cage.

We note that the SM model has the advantage using data from all three isomers at once, rather than relying on fits to the separate pairwise equilibria, each with its own statistical uncertainty.

7 Additional spectroscopic data

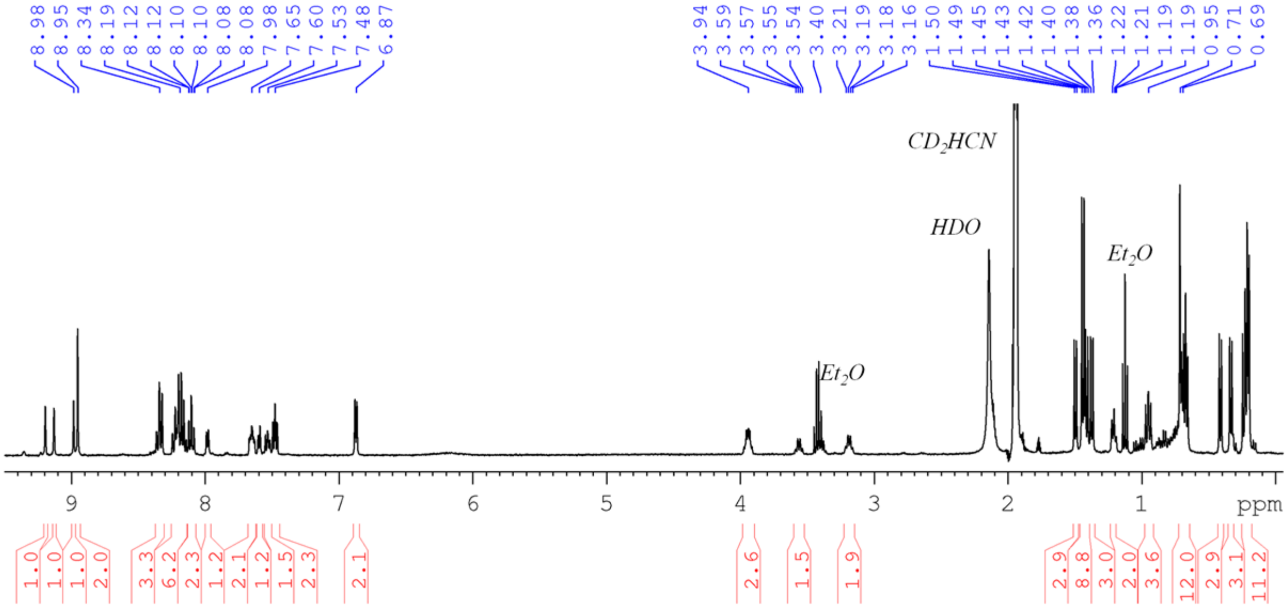

Figure S19. ^{1}H NMR (400 MHz) spectrum of complex **1c** in CD_3CN at room temperature.

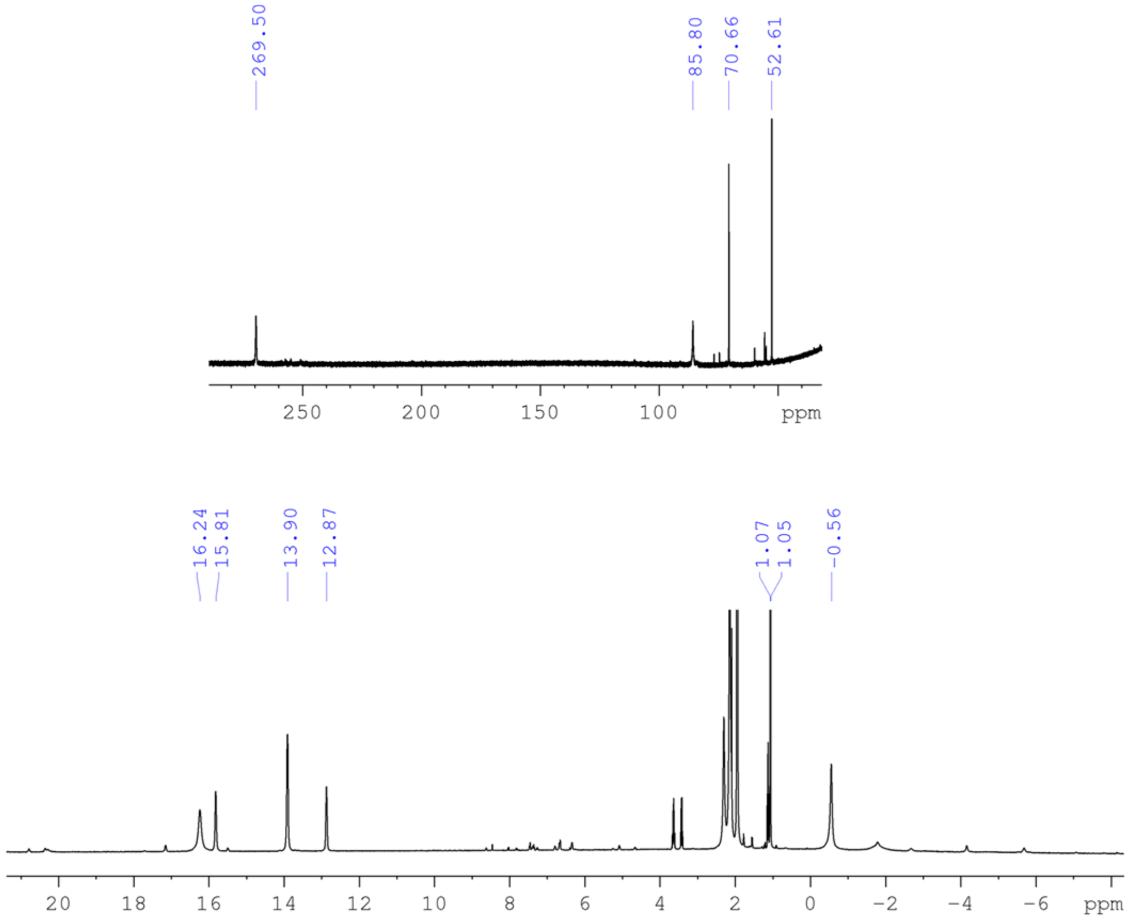

Figure S20. Relevant regions of the ^{1}H NMR (400 MHz) spectrum of complex **3b** in CD_3CN at room temperature.

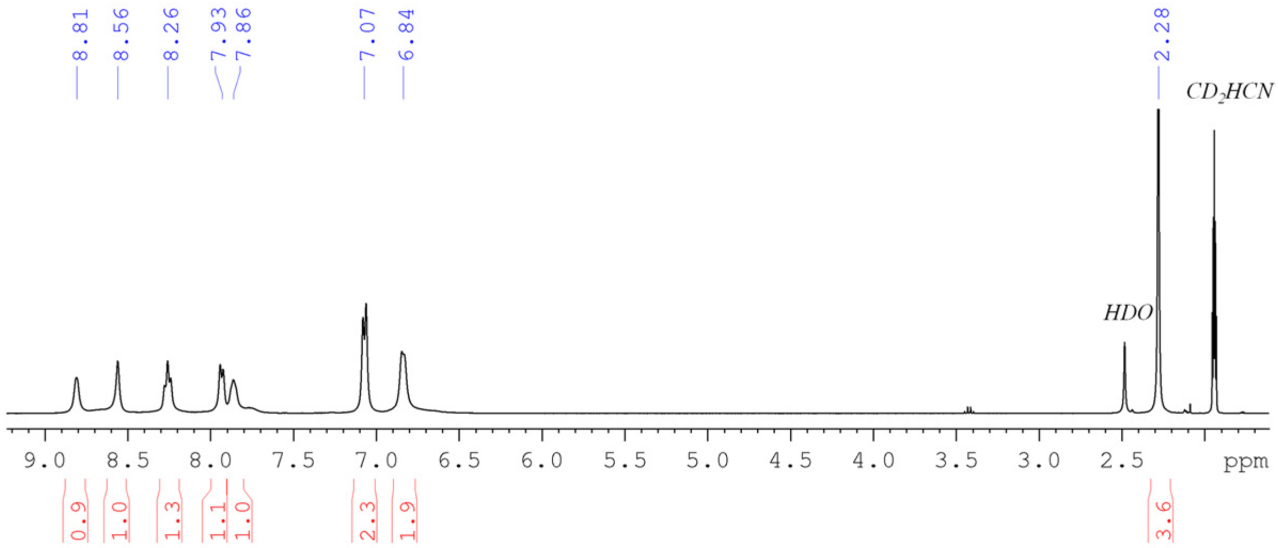

Figure S21. ^{1}H NMR (400 MHz) spectrum of complex **4a** in CD_3CN at room temperature.

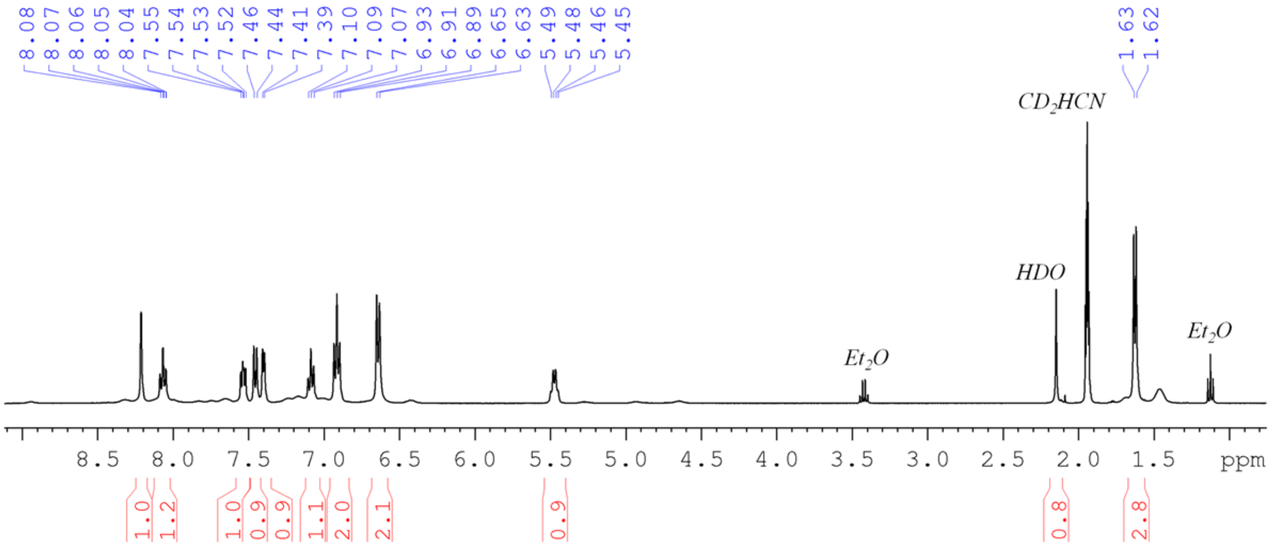

Figure S22. ^{1}H NMR (400 MHz) spectrum of complex **4b** in CD_3CN at room temperature.

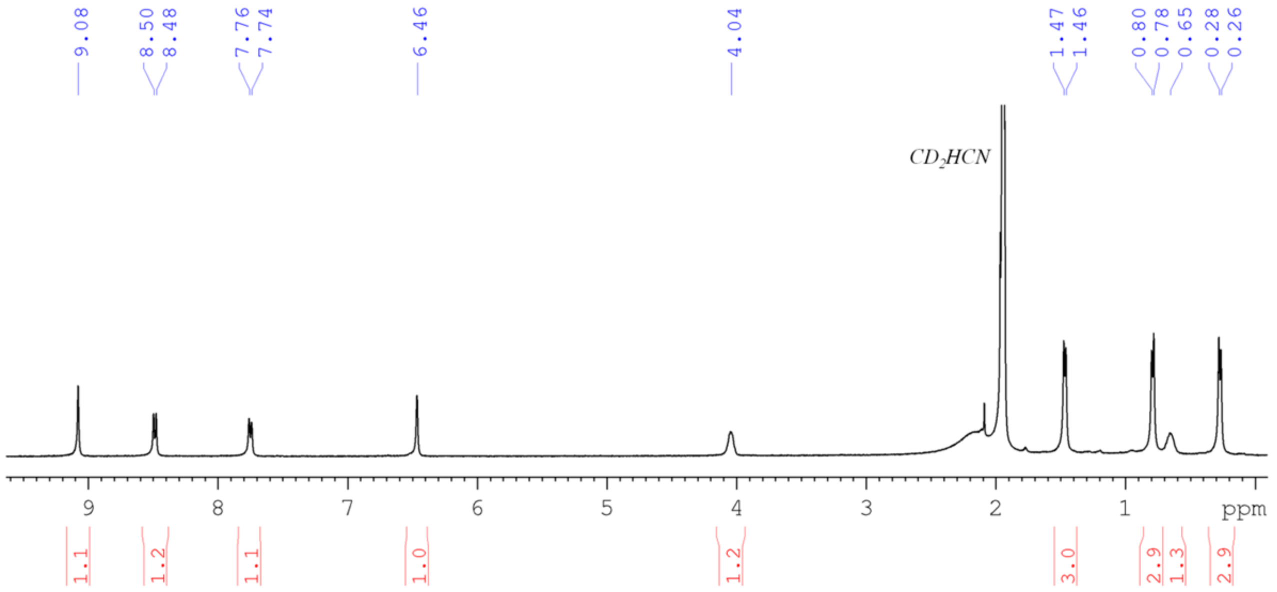

Figure S23. ^{1}H NMR (400 MHz) spectrum of cage **2c**·[OTf]$_8$ in CD_3CN at room temperature.

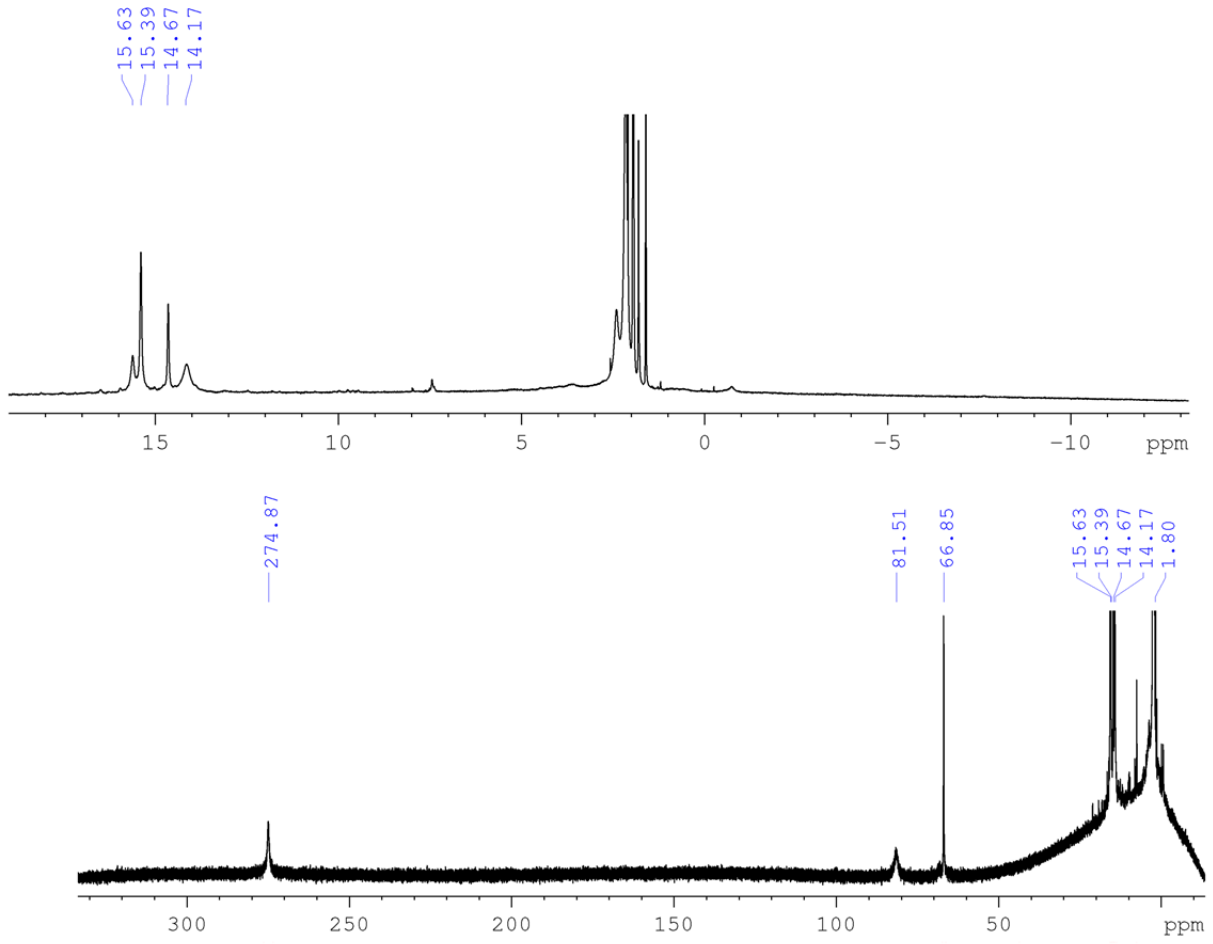

Figure S24. Relevant regions of the ^{1}H NMR (400 MHz) spectrum of complex **5b** in CD_3CN at room temperature.

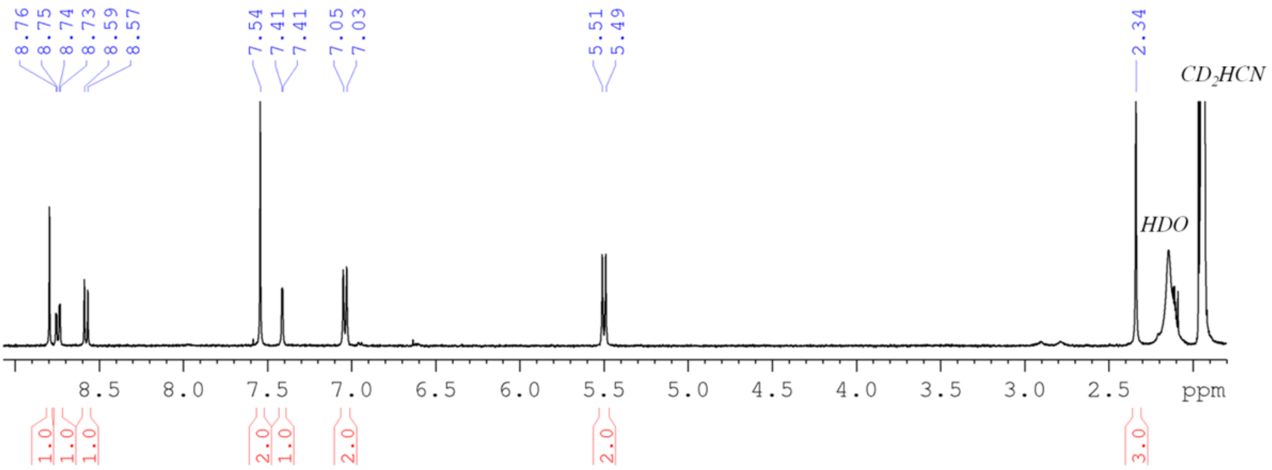

Figure S25. ^{1}H NMR (400 MHz) spectrum of cage **6a**·[OTf]$_8$ in CD_3CN at room temperature.

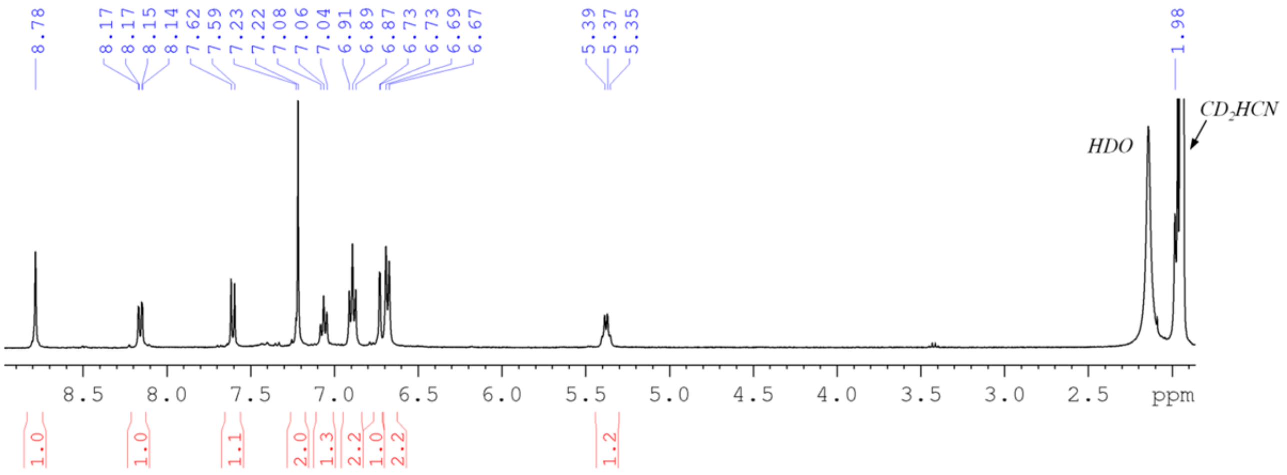

Figure S26. ^{1}H NMR (400 MHz) spectrum of cage **6b**·[OTf]$_8$ in CD_3CN at room temperature.

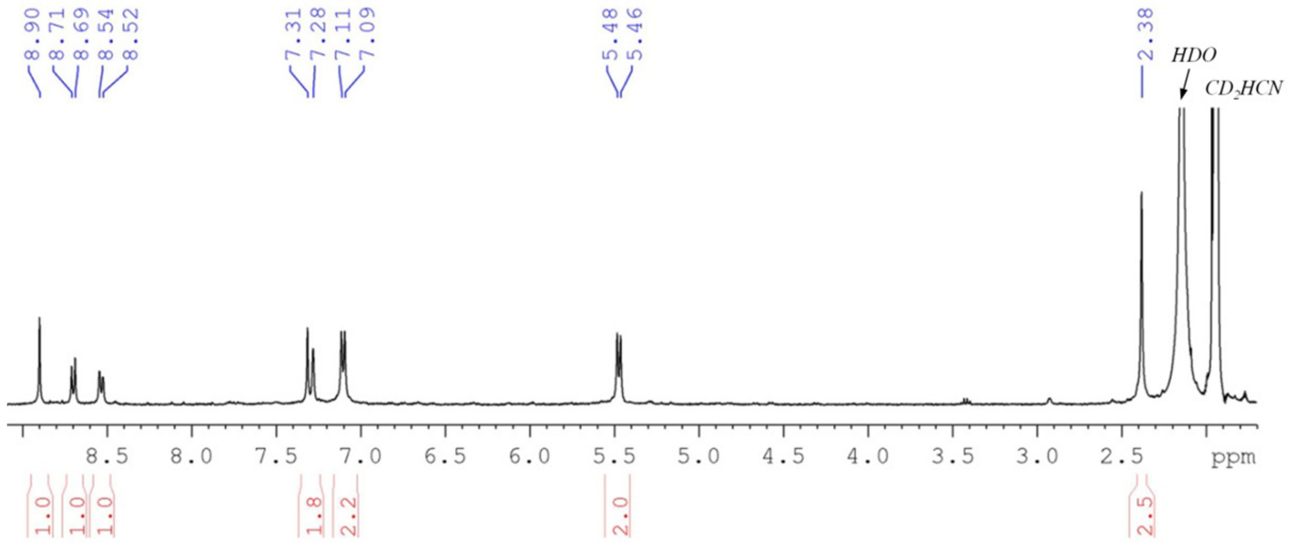

Figure S27. ^{1}H NMR (400 MHz) spectrum of cage **7a**·[OTf]$_8$ in CD_3CN at room temperature.

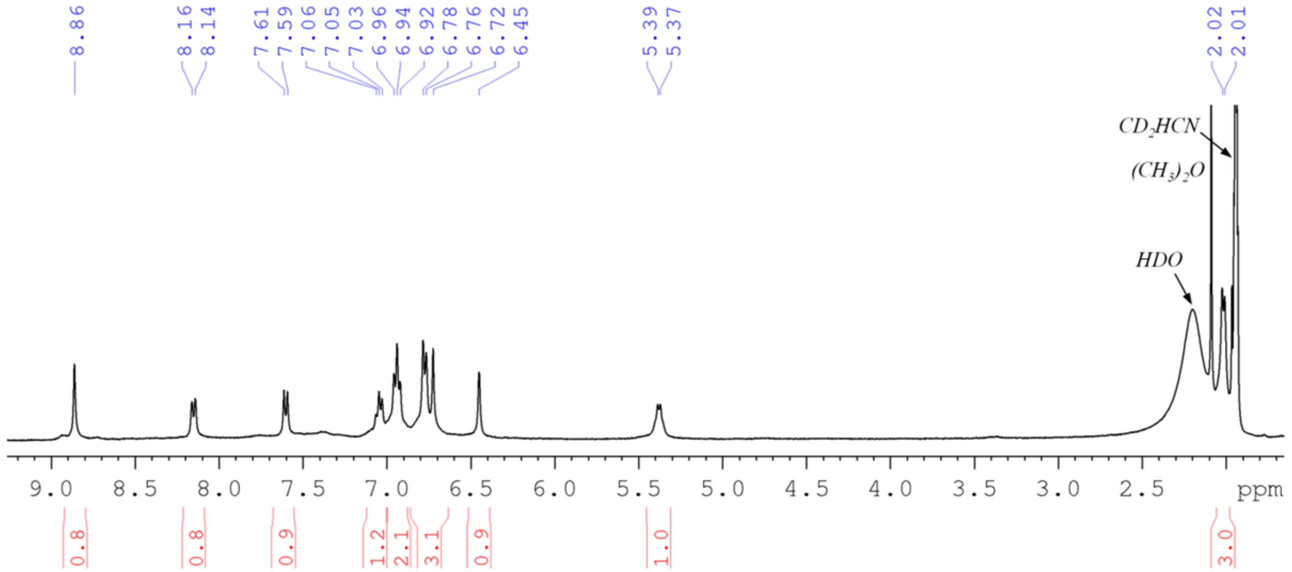

Figure S28. ^{1}H NMR (400 MHz) spectrum of cage **7b**·[OTf]$_8$ in CD_3CN at room temperature.

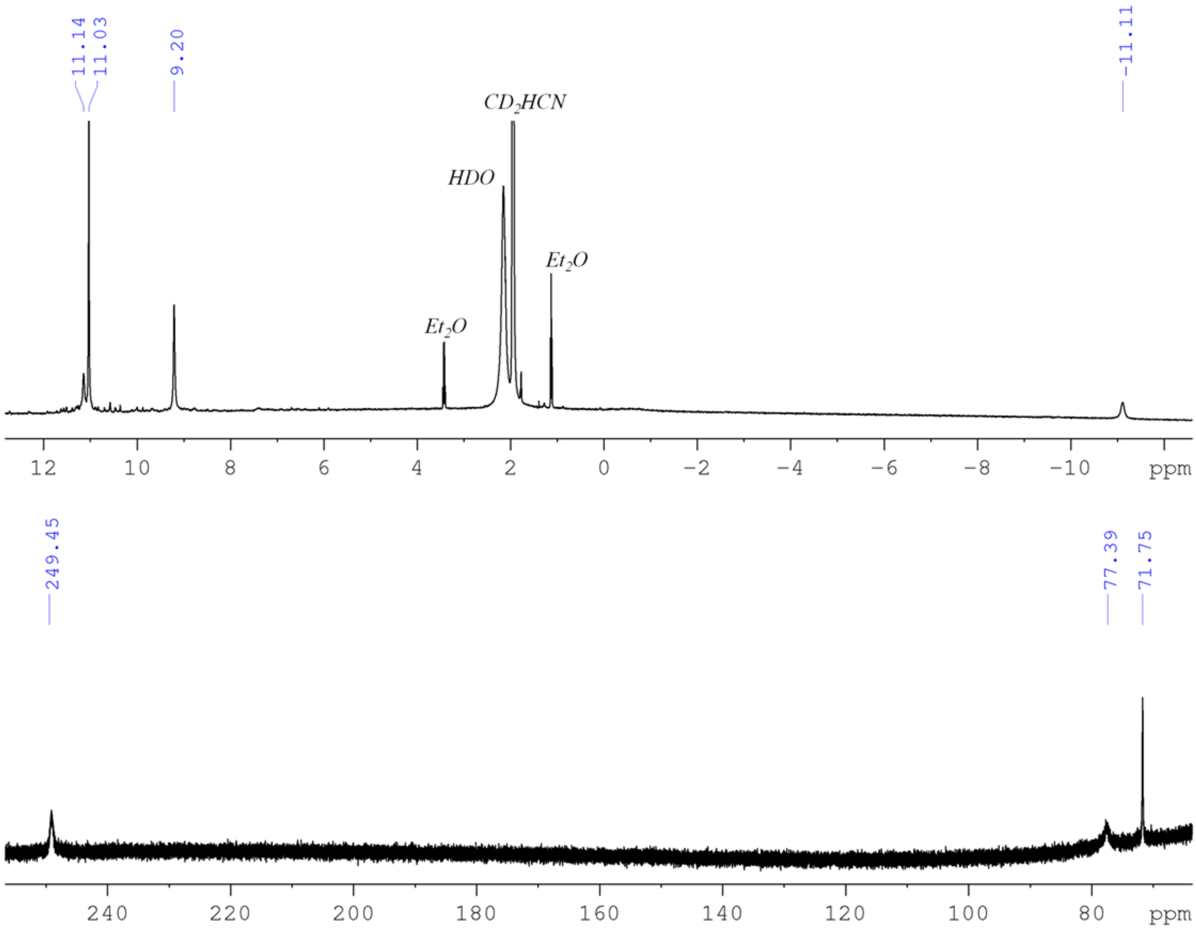

Figure S29. Relevant regions of the ^{1}H NMR (400 MHz) spectrum of cage **8a**·[OTf]$_8$ in CD_3CN at room temperature.

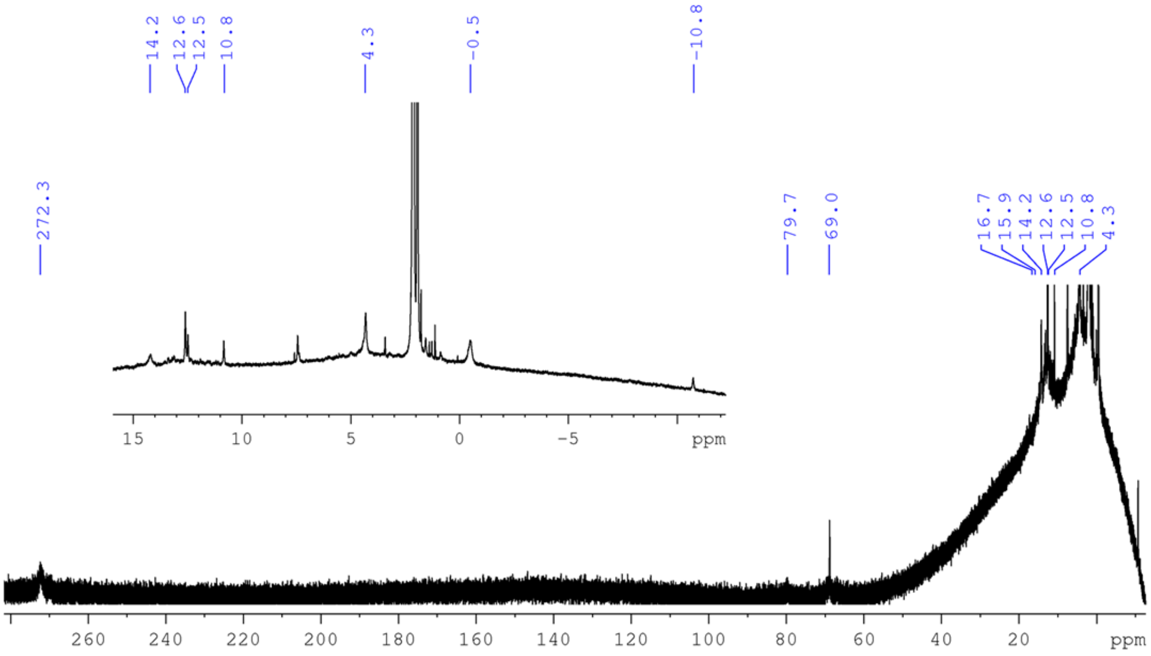

Figure S30. Relevant regions of the ^{1}H NMR (400 MHz) spectrum of cage **8b**·[OTf]$_8$ in CD_3CN at room temperature.

NMR and ESI-MS spectra as representative examples for all Sergeant-and-Soldiers experiments with mononuclear complexes 1, 3 and 4, and cages 2, 5 and 6 (see also Ousaka et al.[S4]):

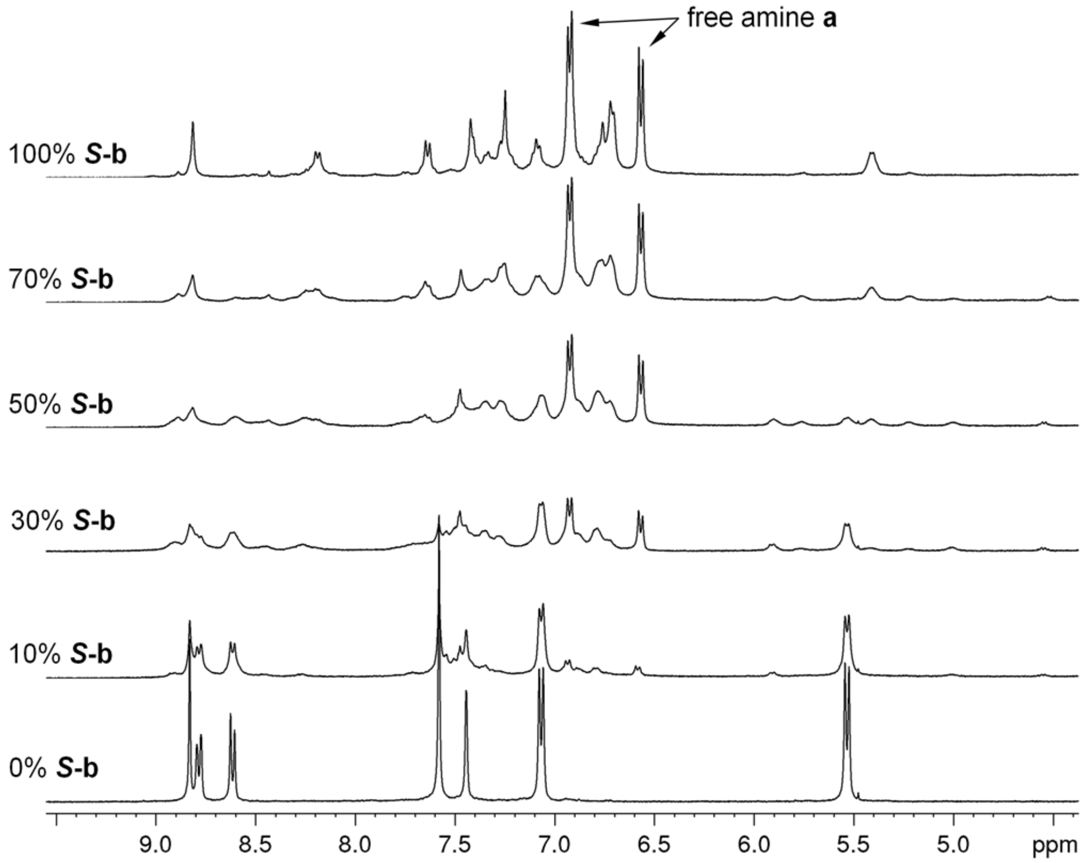

Figure S31. ^{1}H NMR (400 MHz) spectra of cage **6a** in the presence of different amount of ***S*-b** in CD_3CN: [**6a**]$_0$ = 1.0 × 10^{-3} M, [**a**]/[***S*-b**] × 100 = 0, 10, 30, 50, 70 and 100%. The spectra were taken after heating at 70 °C for 2 days and then at room temperature for 1 day. Longer heating produced no further changes in the spectra.

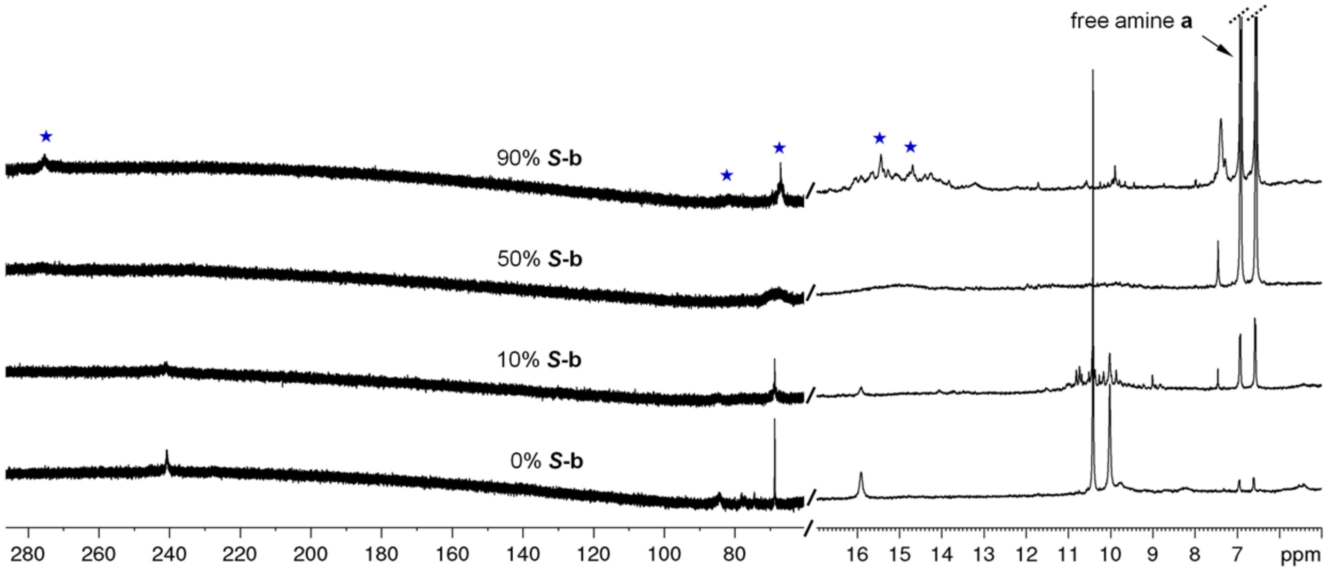

Figure S32. Relevant sections of the ^{1}H NMR (400 MHz) spectra of cage **5a** in the presence of different amount of ***S*-b** in CD_3CN: [**5a**]$_0$ = 9.5 × 10^{-4} M, [**a**]/[***S*-b**] × 100 = 0, 10, 50 and 90%. The spectra were taken after heating at 70 °C for 2 days and then at room temperature for 1 day. Longer heating produced no further changes in the spectra. Intensities have been scaled for clarity. ★ denotes some of the peaks corresponding to cage **5b**.

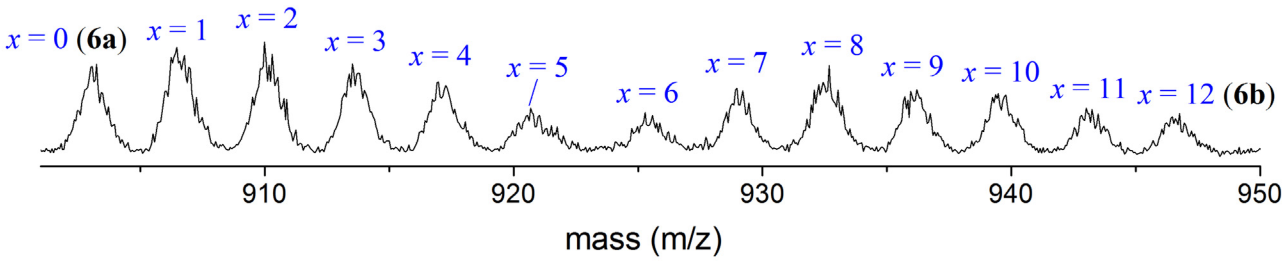

Figure S33. ESI-MS spectrum (+4 peak) of **6a** with 6 equiv of ***S*-b** after heating for 2 days at 70 °C and 1 day at room temperature. x = number of ***S*-b** residues incorporated in the $Fe^{II}_4L_6$ framework substituting **a** residues, where x = 0 corresponds to cage **6a** and x = 12 to **6b**.

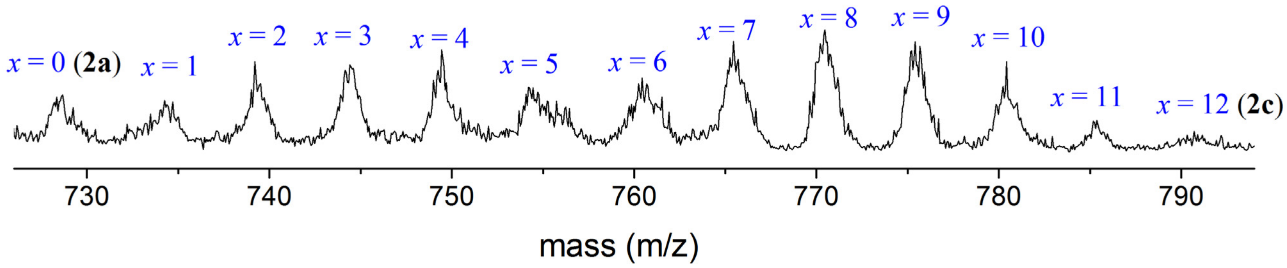

Figure S34. ESI-MS spectrum (+4 peak) of **2a** with 6 equiv of ***S*-c** after heating for 2 days at 70 °C, and then for 1 day at room temperature. x = number of ***S*-c** residues incorporated in the $Fe^{II}_4L_6$ framework substituting **a** residues, where x = 0 corresponds to cage **2a** and x = 12 to **2c**.

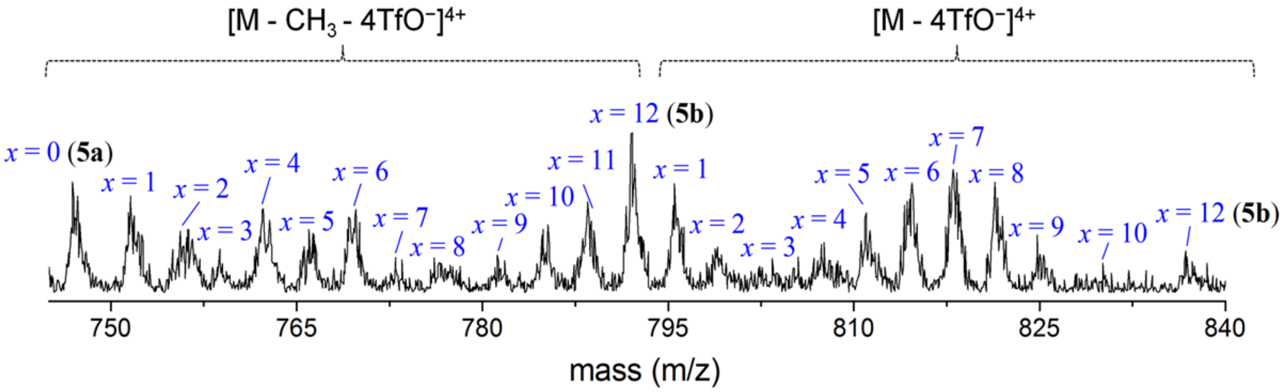

Figure S35. ESI-MS spectrum (+4 peak) of **5a** with 6 equiv of ***S*-b** after heating for 2 days at 70 °C, and then for 1 day at room temperature. *x* = number of ***S*-b** residues incorporated in the $Co^{II}_4L_6$ framework substituting **a** residues, where *x* = 0 corresponds to cage **5a** and *x* = 12 to **5b**. Significant fragmentation in the gas phase was observed with this sample, and in general with the Co^{II} complexes reported here, under the ionization conditions used.

^{1}H NMR and ESI-MS spectra for the Sergeant-and-Soldiers experiment with $Co^{II}_4L_4$ (8a) and S-b.

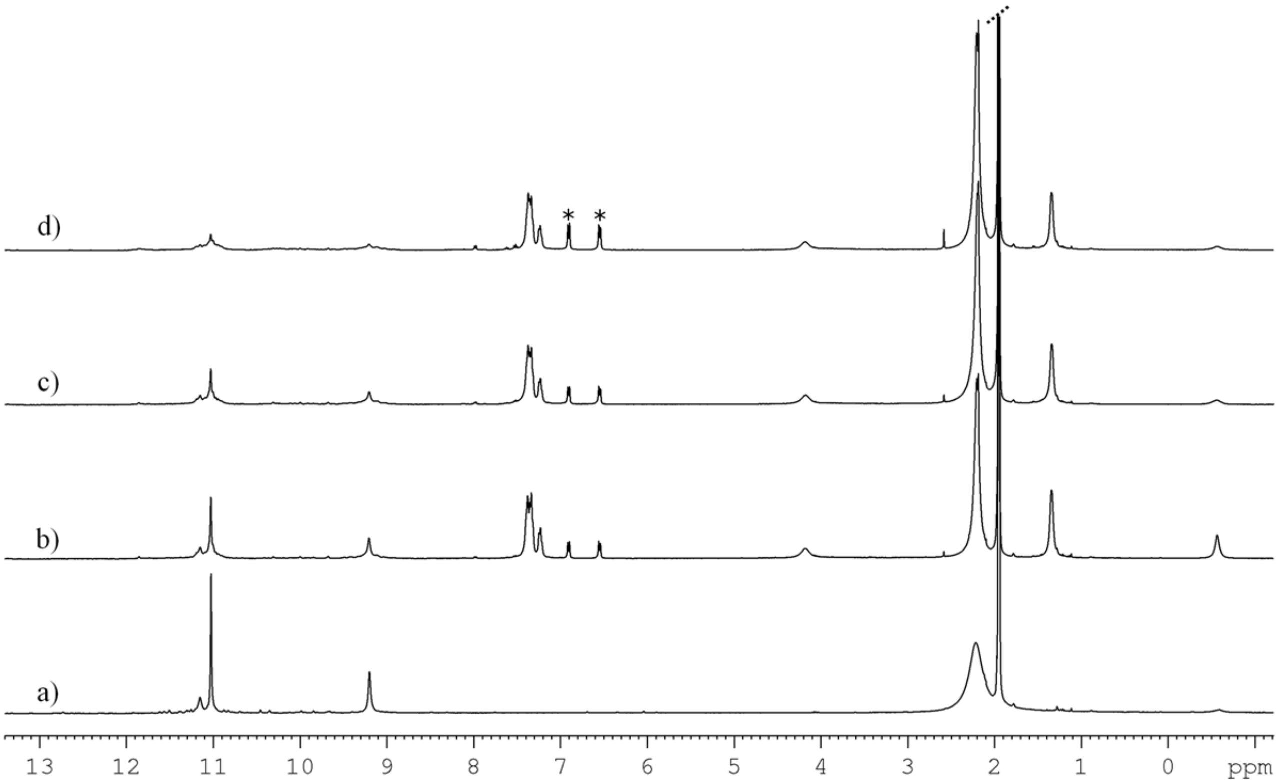

Figure S36. Partial ^{1}H NMR (400 MHz, 298 K, CD_3CN) spectra of a) cage **8a** and b–d) cage **8a** during the course of its reaction with ***S*-b** in CD_3CN: $[\mathbf{8a}]_0 = 1.0 \times 10^{-3}$ M, $[\mathbf{8a}]_0/[\boldsymbol{S}\text{-}\mathbf{b}]_0 = 1/12$. The spectra were taken after heating at 70 °C for b) 4 days, c) 7 days and d) 12 days. Although in spectrum d) small signals corresponding to cage **8a** can be discerned, longer heating produced no further changes in the spectrum. ✳ denotes the peaks corresponding to free amine **a**.

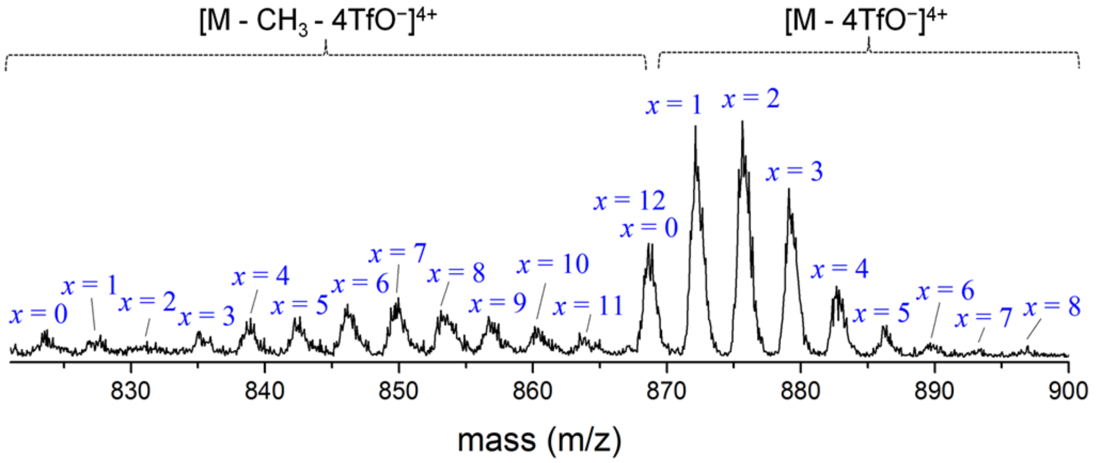

Figure S37. ESI-MS spectrum (+4 peak) of **8a** with 6 equiv of ***S*-b** after heating for 12 days at 70 °C, and then for 1 day at room temperature. *x* = number of ***S*-b** residues incorporated in the $Co^{II}_4L_4$ framework substituting **a** residues, where *x* = 0 corresponds to cage **8a** and *x* = 12 to **8b**. Significant fragmentation in the gas phase was observed with this sample under the ionization conditions used.

8 References

[S1] I. A. Riddell, T. K. Ronson, J. K. Clegg, C. S. Wood, R. A. Bilbeisi, J. R. Nitschke, *J. Am. Chem. Soc.* **2014**, *136*, 9491-9498.

[S2] S. Ma, M. M. J. Smulders, Y. R. Hristova, J. K. Clegg, T. K. Ronson, S. Zarra, J. R. Nitschke, J. R. *J. Am. Chem. Soc.* **2013**, *135*, 5678-5684.

[S3] A. M. Castilla, T. K. Ronson, J. R. Nitschke, *J. Am. Chem. Soc.* **2016**, *138*, 2342-2351.

[S4] N. Ousaka, J. K. Clegg, J. R. Nitschke, *Angew. Chem. Int. Ed. Engl.* **2012**, *51*, 1464-1468.

[S5] I. A. Riddell, M. M. J. Smulders, J. K. Clegg, Y. R. Hristova, B. Breiner, J. D. Thoburn, J. R. Nitschke, *Nat. Chem.* **2012**, *4*, 751-756.

[S6] Y. R. Hristova, M. M. J. Smulders, J. K. Clegg, B. Breiner, J. R. Nitschke, *Chem. Sci.* **2011**, *2*, 638-641.

[S7] A. M. Castilla, N. Ousaka, R. A. Bilbeisi, E. Valeri, T. K. Ronson, J. R. Nitschke, *J. Am. Chem. Soc.* **2013**, *135*, 17999-18006.

[S8] W. Meng, J. K. Clegg, J. D. Thoburn, J. R. Nitschke, *J. Am. Chem. Soc.* **2011**, *133*, 13652-13660.

[S9] I. A. Riddell, T. K. Ronson, J. R. Nitschke, *Chem. Sci.* **2015**, *6*, 3533-3537.

[S10] S. E. Howson, L. E. N. Allan, N. P., Chmel, G. J. Clarkson, R. J. Deeth, A. D. Faulkner, D. H. Simpson, P. Scott, *Dalton. Trans.* **2011**, *40*, 10416-10433.

[S11] C. Piguet, *Chem. Commun.* **2010**, *46*, 6209-6231.

[S12] a) M. Borkovec, G. J. M. Koper, C. Piguet, *Curr. Opin. Colloid Interface Sci.* **2006**, *11*, 280-289; b) G. Koper, M. Borkovec, *J. Phys. Chem. B* **2001**, *105*, 6666-6674.

[S13] G. Ercolani, L. Schiaffino, *Angew. Chem. Int. Ed.* **2011**, *50*, 1762-1768.

[S14] Wolfram Research, Inc., Mathematica, Version 8.0, Champaign, IL (2010).

[S15] D. J. Farrington, J. O. Jones, M. V. Twigg, *Inorg. Chim. Acta* **1977**, *25*, L75-L76.

[S16] J.-F. Ayme, J. E. Beves, D. A. Leigh, R. T. McBurney, K. Rissanen, D. Schultz, *J. Am. Chem. Soc.* **2012**, *134*, 9488-9497.

[S17] S. R. Batten, K. S. Murray, N. J. Sinclair, *Acta Crystallogr., Sect. C* **2000**, *56*, e320.

[S18] Q. Huang, L. Diao, C. Zhang, F. Lei, *Molecules* **2011**, *16*, 2871-2883.

[S19] G. H. Eom, H. M. Park, M. Y. Hyun, S. P. Jang, C. Kim, J. H. Lee, S. J. Lee, S.-J. Kim, Y. Kim, *Polyhedron* **2011**, *30*, 1555-1564.
